# Quantitative Trait Loci for Light Sensitivity, Body Weight, Body Size, and Morphological Eye Parameters in the Bumblebee, *Bombus terrestris*

**DOI:** 10.1371/journal.pone.0125011

**Published:** 2015-04-30

**Authors:** Kevin Maebe, Ivan Meeus, Jan De Riek, Guy Smagghe

**Affiliations:** 1 Department of Crop Protection, Faculty of Bioscience Engineering, Ghent University, Coupure links 653, B-9000, Ghent, Belgium; 2 Institute for Agricultural and Fisheries Research (ILVO), Plant Sciences Unit, Caritasstraat 21, B-9090, Melle, Belgium; University of North Carolina, Greensboro, UNITED STATES

## Abstract

Bumblebees such as *Bombus terrestris* are essential pollinators in natural and managed ecosystems. In addition, this species is intensively used in agriculture for its pollination services, for instance in tomato and pepper greenhouses. Here we performed a quantitative trait loci (QTL) analysis on *B*. *terrestris* using 136 microsatellite DNA markers to identify genes linked with 20 traits including light sensitivity, body size and mass, and eye and hind leg measures. By composite interval mapping (IM), we found 83 and 34 suggestive QTLs for 19 of the 20 traits at the linkage group wide significance levels of *p* = 0.05 and 0.01, respectively. Furthermore, we also found five significant QTLs at the genome wide significant level of *p* = 0.05. Individual QTLs accounted for 7.5-53.3% of the phenotypic variation. For 15 traits, at least one QTL was confirmed with multiple QTL model mapping. Multivariate principal components analysis confirmed 11 univariate suggestive QTLs but revealed three suggestive QTLs not identified by the individual traits. We also identified several candidate genes linked with light sensitivity, in particular the *Phosrestin-1-like* gene is a primary candidate for its phototransduction function. In conclusion, we believe that the suggestive and significant QTLs, and markers identified here, can be of use in marker-assisted breeding to improve selection towards light sensitive bumblebees, and thus also the pollination service of bumblebees.

## Introduction

Bumblebees are essential pollinators in natural and managed ecosystems [1–2]. Several bumblebee species, such as the buff-tailed bumblebee *Bombus terrestris* L., are used worldwide in greenhouses for the pollination of different crops [3]. In the artificial light environment of a greenhouse bumblebees perform better than honeybees (*Apis mellifera*). However, when the artificial light environment of a greenhouse deviates from the natural light environment in intensity and spectral composition, bumblebees also have troubles finding their way back to the colony and have decreased foraging activity [4–8].

Bumblebee performance in greenhouses with artificial light could be enhanced by selection towards more light sensitive bumblebees. One rearing strategy could be simple morphology-based selection towards bigger bumblebees. Larger bumblebees have bigger eyes which should have better light perception and thus should be more light sensitive [9–10]. Indeed, an increase in the size of the morphological parameters of the sensory system enhances the ability to detect and discriminate between different flowers which in turn can increase foraging efficiency [11]. Maebe et al. [12] found that at both intra and inter colony levels, larger *B*. *terrestris* individuals had larger eyes. However, some colonies containing smaller bumblebees also had better light perception compared to colonies with larger specimens. Thus, a large body size did not necessarily correlate with greater light sensitivity or increase foraging efficiency in weak light conditions. Indeed, other morphological parameters, such as larger photoreceptors (rhabdomeres), better molecular photon capture, signal transduction and neuronal composition can play a more important role in optimizing light perception [12] as has also been discussed by Warrant [13] and Kapustjanskij et al. [9].

An alternative strategy could be a marker based selection for more light sensitive bumblebees. For marker-assisted selection (MAS) we need to identify at least one marker linked to the gene or genes responsible for light sensitivity [14–15].

Identification of markers linked with the genes responsible for the phenotypic variation of a certain trait can be determined by quantitative trait loci (QTL) analysis [16–18]. The first step in a QTL analysis is the construction of a genetic linkage map [16]. In social Hymenoptera, like *B*. *terrestris*, a genetic linkage map can be easily constructed as the queen’s meiotic recombination rates can be reliably measured from her male offspring (drones) [17–21]. For *B*. *terrestris* several linkage maps have already been constructed [19–21]. Stolle et al., [21] created a second generation linkage map which showed 18 linkage groups (LGs) with a total length of 2047cM, representing the 18 chromosomes of haploid bumblebee males [22]. QTLs and epistatic interactions have been discovered for several important traits related to immune defence, reproduction [18], host-parasite interactions and body size of *B*. *terrestris* [17].

Here, we performed a QTL analysis on drones of *B*. *terrestris* to determine QTL regions, and to identify markers and epistatic interactions linked with light sensitivity and body size. To this end, we measured the light sensitivity under both blue and UV light conditions of each drone, as well as body size, body mass and several other morphological parameters of the eye and the hind leg for each individual. Furthermore, we genotyped each drone using 136 microsatellite markers. The QTLs and markers identified here show the first promise to be used in marker assisted breeding to improve selection for light sensitive bumblebees.

## Methods

### Mapping population

**Fig 1:**
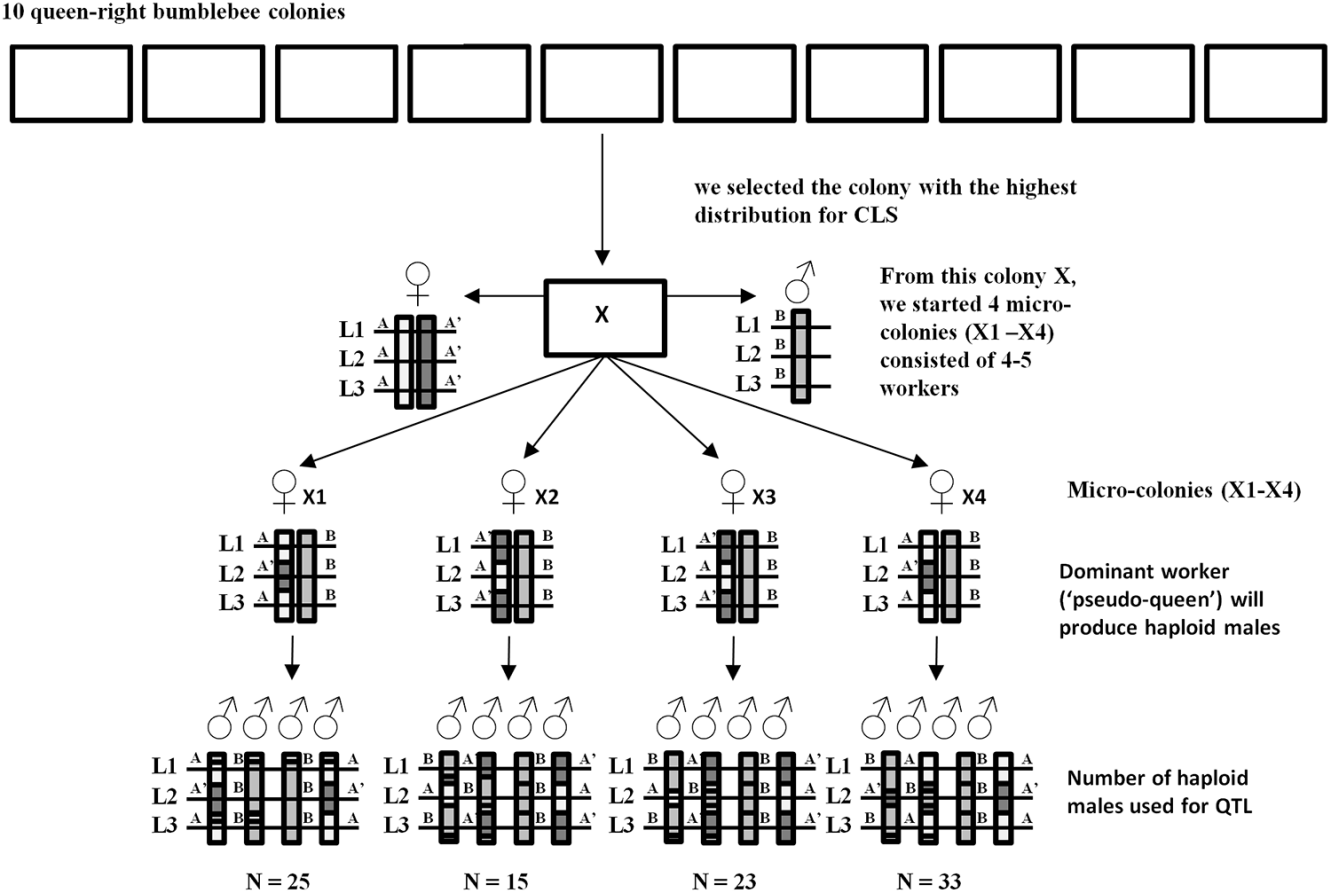
Genetic mapping population. From 10 queen-right bumblebee colonies we selected 1 colony (X). Four micro-colonies were developed with 4–5 workers of colony X (X1-X4). The unfertilized eggs (haploid males) produced by the ‘pseudo-queen’ of these micro-colonies were used for the QTL analysis. In addition, the heritability of three hypothetical loci (L1-L3) are shown, base on the maternal alleles (A and A’) of the queen in colony X, and the paternal allele B of the drone the queen of colony X has mated with.

For this project we received 10 commercial queen-right colonies of *B*. *terrestris* from a mass-rearing program (Biobest, Westerlo, Belgium). From each colony we randomly selected 10 workers and determined their critical light sensitivity (CLS), the lowest light intensity at which an individual bumblebee is able to fly, as described in Maebe et al. [12]. From the colony with the most variation in CLS, we selected additional workers with whose we created 4 micro-colonies consisting of 5 workers each. Micro-colonies are nests made of a small group of new-born worker bees. Within 2 days, one worker becomes dominant, i.e. pseudo-queen, and starts laying unfertilized or haploid eggs that develop into drones while the other workers take care of the brood. The pace of colony development follows a well-defined pattern (i.e., time until first oviposition, first larvae developed, and first pupae) for colonies receiving the same diet *ad libitum* [23,24]. The 96 drones produced by these 4 micro-colonies were used for genetic linkage mapping (Fig 1). All queen-right colonies and micro-colonies were provided with commercial sugar water (BioGluc; Biobest, Westerlo, Belgium) and pollen (Apihurdes, Cáceres, Spain) *ad libitum* in a controlled laboratory environment at 28–30°C and 60–65% air humidity and in continuous darkness.

### Critical light intensity in blue and ultraviolet light

For each drone we determined, under blue and ultraviolet (UV) light conditions, the lowest light intensity at which it is able to fly, applying the bioassay for determination of CLS described in Kapustjanskij et al. [9] and Maebe et al. [12], with some small modifications. An individual drone was placed on a platform (9 cm in diameter) and exposed to blue or UV light. For the blue light condition we positioned a JC-G4 W/20 lamp at 55 cm above the platform and in front of the lamp we placed a Tokyo Blue LEE colour filter (Phlippo Showlights, Lier, Belgium) allowing the transmission of light in the blue spectrum (400–500 nm) together with a LEE UV filter (Phlippo Showlights) to ensure no transmission of UV light. For the ultraviolet light condition, we used a Mini-Lynx 20W BL350 lamp (Havells Sylvania, Tienen, Belgium) allowing the transmission of UV light between 315 and 400 nm with a peak at 352 nm. LEE Neutral Density filters of 0.15, 0.3, 0.6, 0.9 and 1.2 (Phlippo Showlights) were used to reduce the light intensity without altering the spectral composition of the light. Light intensities were measured at the centre of the platform with a calibrated luxmeter (Taschen-Luxmeter LM37, Karlsruhe, Germany). When the drone, could lift up from the platform towards the light, he was scored as “flying”. If a bumblebee could fly at least 3 out of 5 times, the light intensity was lowered with a dimming device (EMD200, Elix). If she could not, she was first encouraged to fly with the help of tweezers to be sure and to make the difference between bumblebee which cannot fly under this conditions or ones which just do not want to fly, and then the light intensity was increased. These steps were repeated until we found the lowest intensity at which she was still able to fly. For further analyses, the CLS values were log transformed.

### Morphological characteristics

For each drone we measured several parameters related to body size and eye morphology as described by Maebe et al. [12]: total fresh body mass; forewing radial cell length; dorsal-ventral length of compound eye, width of compound eye, total surface of compound eye, diameter of facet, total numbers of ommatidia of compound eye, and diameter of median ocellus; and length of hind leg, trochanter length, trochanter width, femur length, femur width, tibia length, tibia width, metatarsus length, metatarsus width, and tarsus length.

The left compound eye from each bumblebee was photographed with a Leica DFC295 digital camera mounted on a Leica S6D microscope (Leica Microsystems Ltd, Switzerland) by using the software LAS vs 3.6.0 (Leica Application Suite). The measurements of all eye parameters were done on these images with the free software program Image J (http://rsb.info.nih.gov/ij/index.html) [25]. The total surface of the compound eye was estimated with the formula of an ellipse surface as described by Maebe et al. [12]. The diameter of a facet was calculated as the mean of a row of 10 facets measured in three dimensions (w, y and z) as described by Kapustjanskij et al. [9]. This was always measured at the centre of the compound eye [12]. The ommatidia surface, a hexagon, was calculated using the formula S = 3√3/2*z^2^ with z as the radius of the ommatidia. Ommatidia numbers were then estimated by dividing the eye surface with the ommatidia surface.

The right forewing and hind leg of each drone were dissected from the body, taped on a transparent paper, and scanned to allow measurements of the wing and different leg parameters with Image J [25]. The forewing radial cell length was considered as representative for bumblebee size as radial cell length correlates well with head width, body mass and wing length [26–27].

### Correlations

Correlations between the different morphological characters were tested by the Spearman correlation test in SPSS (version 22.0.0.0). Instead of the more conservative sequential Bonferroni corrections for multiple significance tests [28], we calculated the false discovery rate by the Benjamini and Hochberg [29] formula [*P*(i) ≤ (*α* * i)/m], with *α* being the significance threshold value, m the number of performed tests and i the number of null hypotheses arranged by ascending *P*-values. Instead of the significance threshold of α = 0.05, we created with this formulae a ‘new threshold value’ for rejection of the null hypothesis, and this for the first i-value which has a lower calculated *P*-value than *P*(i). To achieve this, we searched for the first *P*-value which follows this formula. Here, with *α* = 0.05 and m = 190, we compared each *P*(i) with 0.05(i)/190, starting from *P*(190). As *P*(156) = 0.034 < (0.05*156)/190 our new significance threshold was 0.041. For datasets with many correlated traits, multivariate methods, like PCA, are often performed to reduce the dimensionality of the dataset without losing much of the original variation [30]. Thereby, the principal components (PCs) can serve as traits in the QTL analysis [30]. Here, we performed a PCA for the different body size traits and also for the different eye traits with Primer 6 [31]. The PCs with the largest eigenvalues were used for PC-QTL mapping.

### DNA extraction and microsatellites protocol

Bumblebee DNA was extracted from one middle leg of each drone as described in Maebe et al. [32]. Bumblebees were genotyped at 131 microsatellite loci developed for *B*. *terrestris*: 12 loci from Stolle et al. [33], 11 loci from Reber-Funk et al. [34], 106 loci developed from a BAC-library [35] by Stolle et al. [21], one new loci by Stolle et al. [21] and one locus from Estoup et al. [36–37] (S1 Table). Additionally, we used 4 loci derived from *B*. *lucorum* [34] and one locus from the honeybee, *Apis mellifera* [38] (S1 Table). All 136 microsatellite loci, used in this study, were already used before to construct a second generation genetic map of *B*. *terrestris* [21].

For detection of the microsatellite alleles, we used a tailed-primer approach [39]: a universal M13-primer (= tail, 5’-GAGTTTTCCCAGTCACGAC-3’) is coupled to a HEX, 6-FAM, VIC or NED fluorescent label to allow detection of the microsatellite alleles by capillary electrophoreses. Furthermore, for incorporation of this universal tail during PCR, the specific forward primers are prolonged at its 5’-end with the same (but unlabeled) sequence as the tail.

Each microsatellite locus was amplified in simplex by PCR. PCR reactions were carried out in 10 μl total volume. Each reaction contained 1.5 μl template DNA, 1 μl of 10x PCR buffer (Qiagen), 0.2 μl of 10 mM dNTP’s (Qiagen), 0.1 μl of 10 μM forward primer, 0.4 μl of 10 μM reverse primer, 0.4 μl of 10 μM labeled M13-primer and 0.05 μl of 2.5 units/reaction Hotstar Taq DNA Polymerase (Qiagen). Samples were initially denatured at 95°C for 15 min, followed by 30 cycles of denaturing at 94°C for 30 s, annealing at 48, 52 or 58°C for 30 s, and extension at 72°C for 30 s. The PCR protocol ended with a final extension step at 72°C for 10 min. After pooling the final PCR products, they were visualized on a ABI-3730xl sequencer (Applied Biosystems) using an internal size standard (Genescan 500 LIZ, Applied Biosystems). The fragments were examined and scored manually using Peak Scanner Software v 1.0 (Applied Biosystems).

### Linkage mapping and phase determination

First, a preliminary linkage mapping was established using 100 microsatellite loci with Kosambi’s mapping function. These loci were chosen based on their known distribution on the 18 linkage groups described in Stolle et al. [21] to obtain an as high as possible cover of the bumblebee genome. The mean number of markers on each linkage group was 5.55 (range: 2–9), with the minimum and maximum distance between two markers ranging between 2.72 cM and 65.56 cM (S2 Table). After identifying different QTL regions with this mapping on 16 linkage groups (LG), we conducted a fine mapping with 36 additional SSR markers, specifically chosen to cover better the preliminary QTL regions. Furthermore, knowing that the bumblebee genome size is 2047.09 cM [21], an power estimation of 136 markers based on the formula c = 1-e^-2md/L^ with m = number of markers, d = distance between markers (in cM), L = genome length and c = proportion of the genome within this distance d, as described in [40] and used in ref. [21], showed that 93.0% of the bumblebee genome is at average located within 20 cM of a marker and 73.5% within 10 cM of a marker.

Linkage analysis was performed with JoinMap software version 4.0 [41]. Linkage groups were estimated by applying independent Logarithm of the Odds (LOD) threshold ranges from 1.0 to 10.0 in steps of 1.0. The initial grouping for mapping was selected from the groupings tree, preferentially by taking (smaller) nodes that showed a stable number of markers at the higher LOD score. We preferred to start from smaller but highly stable linkage groups. Regression linkage maps were established under the standard calculation settings of JoinMap 4.0 (linkages with a recombination frequency smaller than 0.45 and LOD higher than 1; goodness-of-fit jump threshold for removal of loci 5 and performing a ripple after adding one locus). The order of the SSR-markers in our grouping was compared with their order in the second generation linkage map constructed on 577 males of one *B*. *terrestris* colony as described by [21]. Linkage phases were then estimated by JoinMap 4.0.

### QTL analysis

First, we performed the Kruskal-Wallis (KW) test, a single marker non-parametric method imbedded in the software program MapQTL5.0 [42] to detect possible QTLs as done in several other studies (e.g., [25,43]). Secondly, we performed a composite Interval Mapping analysis (IM) with MapQTL 5 [44]. The LOD thresholds for declaring a suggestive QTL (linkage group wide) or a significant QTL (genome wide) were obtained by standard permutation tests (1000 iterations) with MapQTL 5.0 [44] for the significance level *p* = 0.05 and *p* = 0.01. These permutation tests are less dependent on normal distributions to calculate significance thresholds. Third, we performed also a multiple QTL model mapping (MQM) within MapQTL 5.0. The selection of obtained suggestive QTLs in IM were used as cofactors during the MQM-mapping which allowed for the detecting of additional QTLs[17]. When the LOD value of the QTL, assigned as cofactor, dropped during the MQM mapping below the threshold value, then the QTL was removed as cofactor and MQM was run again. We repeated this procedure until the list of cofactors remained stable. For both IM and MQM, the traits need to follow a normal distribution. Most traits were significantly different from normality (S3 Table). However, the Box-Cox transformation had none or only very small effects on the size of the observed QTL regions. For the graphical presentation of the QTLs and markers we employed the software MapChart version 2.2 [45].

### Epistasis analysis

We scanned for epistatic interactions of QTLs using the software QTLMapper version 1.6 [46]. Digenic epistatic effects were tested with both the likelihood ratio (LR) and the *t*-test. Only the most significant interaction was reported when multiple significant epistatic interactions for several marker pairs between the same regions of two linkage groups were found.

### Identification of candidate genes

Candidate genes for light sensitivity were selected around the 95% confidence interval (= C.I.) of the QTL. The two SSR markers which determined the 95% C.I. of the QTL, were found in the bumblebee genome (http://www.ncbi.nlm.nih.gov/genome/2739) and all genes on this sequence (± 500k bp) were selected as candidate genes. We searched in UniProt (http://www.uniprot.org/) for the known function of those candidate genes, and selected the candidate gene which function could be directly linked with vision or light perception as primary target gene.

## Results

### Correlation between traits

In total, 96 drones were measured for 20 different traits (Table 1). The distribution of each of these traits can be seen in S1 Fig There were no indications of significant colony effects (for all traits: Kruskal-Wallis test, *P* > 0.05). Most morphological parameters of the leg and the body size correlated significantly with body mass and the different eye morphology parameters (Table 2). The only two exceptions were: (i) the number of ommatidia did not correlate with facet diameter (*r*_*s*_ = -0.171, *P* = 0.098); and (ii) body mass did not correlate with tibia length and width (*r*_*s*_ = 0.156, *P* = 0.128; *r*_*s*_ = 0.207, *P* = 0.043, respectively), femur width (*r*_*s*_ = 0.146, *P* = 0.157), and both the trochanter length and width (*r*_*s*_ = 0.088, *P* = 0.395; *r*_*s*_ = -0.020, *P* = 0.846, respectively). Furthermore, we detected no correlation between birth order of the males and both bumblebee body size and body weight.

**Table 1:** Means (± S.D.), Skewness and Kurtosis of the investigated traits.

	Code	N	Mean	±SD	Skewness	Kurtosis
Radial cell (cm)	RC	95	0.319	0.035	-0.449	-0.511
Metatarsus length (cm)	MT_L	96	0.285	0.038	-0.564	-0.273
Metatarsus width (cm)	MT_W	96	0.090	0.012	-0.096	0.211
Tibia length (cm)	Ti_L	96	0.429	0.054	-0.705	-0.037
Tibia width (cm)	Ti_W	96	0.119	0.018	0.020	-0.527
Femur length (cm)	Fe_L	96	0.372	0.056	-0.640	-0.276
Femur width (cm)	Fe_W	96	0.124	0.063	5.993	43.288
Trochanter length (cm)	Tr_L	96	0.067	0.012	-0.030	-0.295
Trochanter width (cm)	Tr_W	96	0.091	0.021	-0.889	2.889
Tarsus length (cm)	Tarsus	92	0.585	0.079	-0.665	-0.323
Leg length (cm)	Leg	92	1.452	0.187	-0.619	-0.423
Eye length (mm)	E_L	95	2.554	0.214	-0.994	0.542
Eye width (mm)	E_B	95	1.080	0.088	-1.199	1.298
Facet length (mm)	Facet	94	0.025	0.002	-0.161	-0.052
Median ocellus (mm)	MOc	94	0.279	0.031	-0.436	-0.430
Eye surface (mm^2^)	E_S	95	2.180	0.340	-0.974	0.479
Ommatida number	Om	94	5587	760.7	0.695	1.177
Dry weight (g)	Weight	96	0.211	0.064	0.038	-0.314
CLS under blue light*	CLS_Blue	96	0.431	0.317	0.278	-0.834
CLS under UV light*	CLS_UV	96	0.223	0.117	0.248	-0.454

* after log transformation.

**Table 2:** Correlation coefficients between the investigated traits.

		RC	MT_L	MT_W	Ti_L	Ti_W	Fe_L	Fe_W	Tr_L	Tr_W	Tarsus	Leg	E_L	E_W	Facet	MOc	E_S	Om	Weight	CLS_Blue	CLS_UV
**RC**	r	-																			
**MT_L**	r	0.891**	-																		
**MT_W**	r	0.753**	0.811**	-																	
**Ti_L**	r	0.837**	0.897**	0.835**	-																
**Ti_W**	r	0.679**	0.727**	0.827**	0.860**	-															
**Fe_L**	r	0.794**	0.878**	0.778**	0.868**	0.758**	-														
**Fe_W**	r	0.760**	0.783**	0.732**	0.808**	0.763**	0.792**	-													
**Tr_L**	r	0.412**	0.439**	0.453**	0.504**	0.450**	0.397**	0.391**	-												
**Tr_W**	r	0.572**	0.559**	0.505**	0.597**	0.462**	0.461**	0.592**	0.455**	-											
**Tarsus**	r	0.875**	0.948**	0.827**	0.915**	0.758**	0.877**	0.766**	0.456**	0.552**	-										
**Leg**	r	0.859**	0.938**	0.855**	0.956**	0.826**	0.939**	0.824**	0.518**	0.554**	0.963**	-									
**E_L**	r	0.766**	0.829**	0.766**	0.798**	0.703**	0.825**	0.707**	0.455**	0.471**	0.836**	0.859**	-								
**E_W**	r	0.744**	0.781**	0.699**	0.741**	0.606**	0.744**	0.717**	0.389**	0.439**	0.750**	0.776**	0.807**	-							
**Facet**	r	0.565**	0.579**	0.456**	0.571**	0.530**	0.595**	0.627**	0.316**	0.405**	0.563**	0.598**	0.587**	0.563**	-						
**MOc**	r	0.790**	0.847**	0.785**	0.802**	0.657**	0.833**	0.749**	0.378**	0.508**	0.846**	0.864**	0.779**	0.759**	0.502**	-					
**E_S**	r	0.786**	0.842**	0.766**	0.803**	0.690**	0.828**	0.738**	0.443**	0.456**	0.830**	0.859**	0.958**	0.931**	0.596**	0.802**	-				
**Om**	r	0.443**	0.478**	0.514**	0.422**	0.341**	0.402**	0.305**	0.237*	0.222*	0.487**	0.467**	0.541**	0.552**	-0.171	0.488**	0.564**	-			
**Weight**	r	0.313**	0.280**	0.293**	0.156	0.207	0.260*	0.146	0.088	-0.020	0.276**	0.266*	0.339**	0.326**	0.221*	0.291**	0.349**	0.258*	-		
**CLS_Blue**	r	-0.172	-0.228*	-0.227*	-0.157	-0.238*	-0.086	-0.166	-0.126	-0.042	-0.221*	-0.182	-0.143	-0.082	-0.183	-0.164	-0.112	0.073	-0.398**	-	
**CLS_UV**	r	-0.169	-0.218*	-0.265*	-0.128	-0.241*	-0.080	-0.165	-0.004	-0.096	-0.202	-0.141	-0.192	-0.145	-0.107	-0.191	-0.163	-0.073	-0.388**	0.829**	-

** Correlation is significant at the 0.01 level (2-tailed) after corrections following [29], with as new *P* = 0.008.

* Correlation is significant at the 0.05 level (2-tailed)) after corrections following [29], with as new *P* = 0.041.

The mean critical light sensitivity (CLS), being the lowest light intensity at which a bumblebee is able to fly, of 4 days-old drones (n = 96) in blue and UV light conditions was 3.58 ± 2.89 lux and 1.73 ± 0.47 lux, respectively (Table 1). As light sensitivity could be linked with size parameters [9–12], we searched for correlations between different parameters of bumblebee body size, eye and hind leg with CLS. For most of these morphological parameters we found no significant correlation with the CLS in blue or UV conditions (*P* > 0.041). The CLS in blue and UV light conditions correlated only with the metatarsus length (*r*_*s*_ = -0.228, *P* = 0.025; *r*_*s*_ = -0.218, *P* = 0.033; respectively), the metatarsus width (*r*_*s*_ = -0.227, *P* = 0.026; *r*_*s*_ = -0.265, *P* = 0.009; respectively), and the tibia width (*r*_*s*_ = -0.238, *P* = 0.020; *r*_*s*_ = -0.241, *P* = 0.018; respectively). Furthermore, CLS in blue light sensitivity correlated also with the tarsus length (*r*_*s*_ = -0.221, *P* = 0.034; Table 2).

### QTL analysis

**Fig 2:**
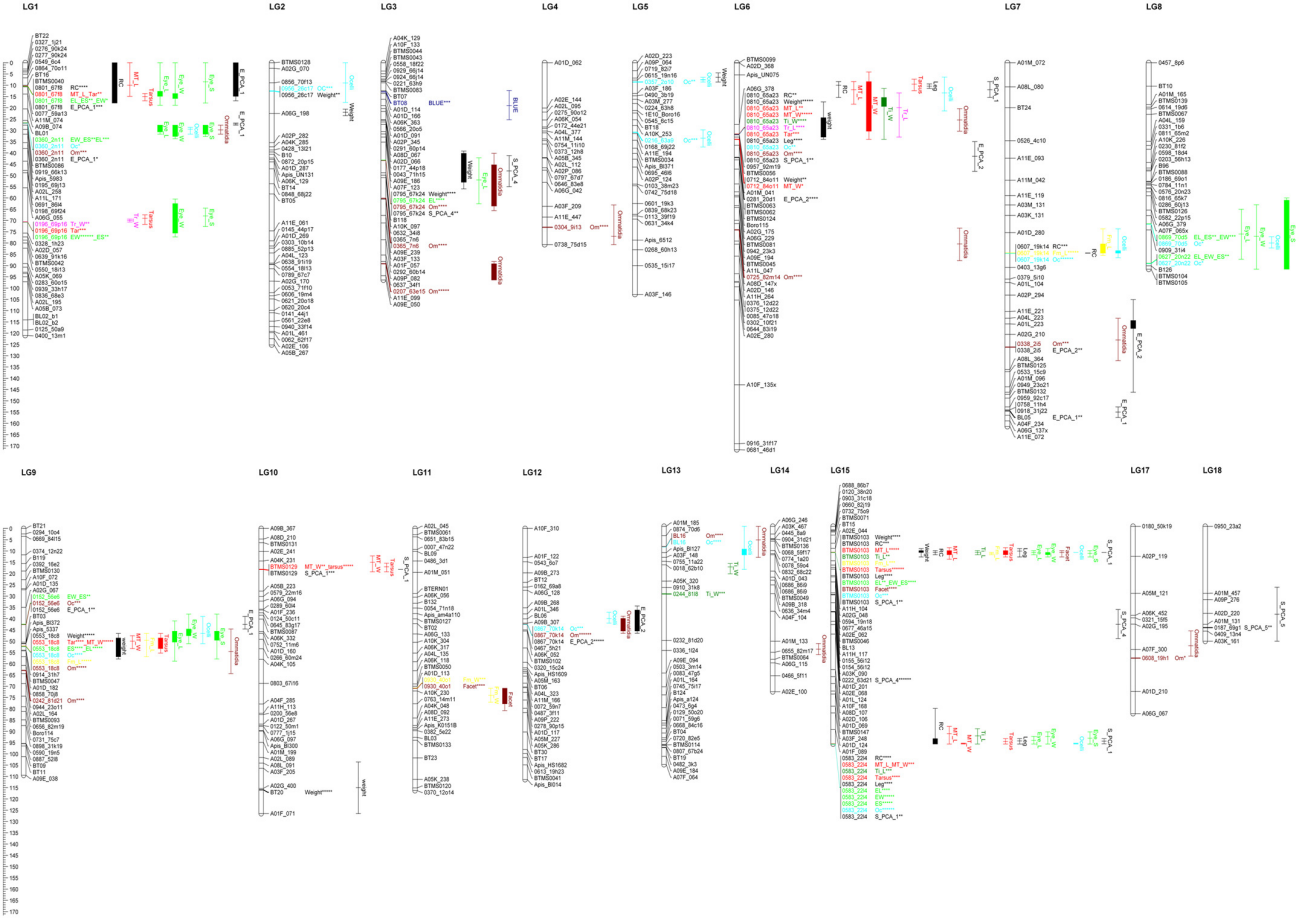
Genetic linkage map showing the distribution of the suggestive and significant QTLs. QTLs for each trait are colour coded: (i) forewing radial cell length(RC), body mass (weight), and length of hind leg (Leg) in black; (ii) metatarsus length (MT_L), metatarsus width (MT_W), and tarsus length (tarsus) in red; (iii) trochanter length (Tr_L), and trochanter width (Tr_W) in fuchsia; (iv) femur length (Fm_L), and femur width (Fm_W) in yellow; (v) tibia length (Ti_L), and tibia width (Ti_W), length of compound eye (E_L), width of compound eye (E_W), and total surface of compound eye (E_S) in green; (vi) diameter of facet (Facet), and total numbers of ommatidia (Om) in maroon; and (vii) diameter of median ocellus (MOc) in light blue. PC-QTLs of the eye parameters and body size are all coloured black: for eye size (E_PCA_1 and E_PCA_2) and for body size (S_PCA1, S_PCA_4 and S_PCA_5). Linkage group number are shown on top of the groups, and map distance (cM) is shown on the left margin of the figure. The genetic map originated from Stoll et al. 2011. The significant markers within suggestive and significant QTL regions are shown with correspondence Kruskal-Wallis significance level (* = 0.10; ** = 0.05; *** = 0.01; **** = 0.005; ***** = 0.001; ****** = 0.0005; and ******* = 0.0001).

Of the 136 SSR markers, 111 were polymorphic across our population (S1 Table). By composite interval mapping (IM) we found 5 significant QTLs and 83 suggestive QTLs for 19 of the 20 traits evaluated (Table 3), with the only exception being for CLS under UV light conditions. Individual QTLs accounted for 7.5–53.3% of the phenotypic variation and were distributed in 16 LGs (Table 3, Fig 2). We found one suggestive QTL for CLS in blue light conditions (*qBLU3*) explaining 10.6% of the genotypic variation, one significant and six suggestive QTLs for body mass, one significant and four suggestive QTLs for radial cell length, one significant and 47 suggestive QTLs for eye traits, and two significant and 26 suggestive QTLs for leg traits (Table 3). Of those 88 QTLs significant at the LG specific or genome wide significance level of 0.05%, 34 QTLs were also significant at the 0.01% LG specific significance level (Table 3).

**Table 3:** List of all identified suggestive QTLs with IM and/or MQM ranked by trait and linkage group (LG).

	Name	Location			IM				MQM				Mean allelic value	
Trait	QTL	LG	Closest marker	KW^a^	LOD^b^	R^2^	0.05^c^	0.01^c^	LOD^a^	R^2^	0.05^d^	0.01^d^	A^e^	B^e^
Radial cell	qRAC1	1	0801_67f8	****	2.41	20.1	0.0–17.9	0.0–17.9	2.52	9.4	10.4–10.4	10.4–10.4	0.30	0.33
	qRAC6	6	0810_65a23	**	2.26	38.7	8.4–15.4	-	-	-	-	-	0.34	0.32
	qRAC7	7	0607_19k14	***	1.57	7.5	84.4–84.5	-	-	-	-	-	0.34	0.31
	qRAC15.1	15	BTMS0103	***	2.06	9.6	10.8–12.6	-	-	-	-	-	0.30	0.33
	qRAC15.2	15	0583_22I4	****	**3.18**	**16.4**	**80.8–96.6**	94.2–96.6	**3.47**	**14.4**	**90.2–96.6**	96.6–96.6	0.34	0.32
Metatarsus length	qMTL1	1	0801_g7f8	**	2.06	16.7	0.0–14.9	-	3.41	14.1	10.4–10.4	10.4–10.4	0.28	0.30
	qMTL6	6	0810_65a23	**	2.42	37.5	8.37–18.4	-	3.09	20.1	27.4–31.8	27.4–31.8	0.32	0.29
	qMTL15.1	15	BTMS0103	*****	3.07	13.7	9.48–14.6	10.8–12.6	-	-	-	-	0.27	0.30
	qMTL15.2	15	0583_22I4	***	2.12	10.2	88.8–96.6	-	-	-	-	-	0.26	0.29
Metatarsus width	qMTW6	6	0810_65a23	*****	**2.98**	**33.4**	**4.09–33.0**	8.37–30.4	**2.74**	**22.0**	**27.4–31.8**	27.4–30.4	0.10	0.09
	qMTW9	9	0553_18c8	*****	1.99	15.6	47.6–53.3	-	-	-	-	-	0.10	0.09
	qMTB10	10	BTMS0129	**	1.79	12.0	12.2–19.2	-	-	-	-	-	0.10	0.09
	qMTL15	15	0583_22I4	***	1.81	8.7	96.2–96.6	-	-	-	-	-	0.08	0.09
Tibia length	qTIL15.1	15	BTMS0103	**	1.84	9.4	10.8–12.6	-	-	-	-	-	0.41	0.44
	qTIL15.2	15	0583_22I4	***	2.10	9.7	89.8–96.6	-	2.10	9.7	91.2–96.6	-	0.38	0.43
Tibia width	qTIW6	6	0810_65a23	****	2.73	53.3	11.4–34.0	15.4–19.4	2.39	17.8	27.4–31.8	-	0.13	0.12
	qTIW13	13	0244_81I8	***	1.54	15.5	16.5–21.1	-	-	-	-	-	0.13	0.12
Femur length	qFML7	7	0607_19k14	*****	2.19	13.3	73.7–85.5	80.4–84.5	2.17	13.0	75.4–86.5	76.4–85.5	0.41	0.36
	qFML9	9	0553_18c8	*****	2.35	22.4	46.6–56.9	49.6–49.6	-	-	-	-	0.41	0.36
	qTIL15	15	BTMS0103	***	2.15	12.3	11.6–12.6	-	2.30	15.8	10.9–14.6	-	0.35	0.38
Femur width	qFMW11	11	0930_40o1	***	2.03	9.3	70.9–70.9	-	2.03	9.3	70.9–70.9	-	0.11	0.12
Trochanter length	qTRL6	6	0810_65a23	****	**2.89**	**35.1**	**13.4–33.0**	-	**2.61**	**23.4**	**27.4–31.8**	27.4–30.8	0.08	0.07
Trochanter width	qTRW1	1	0196_69p16	**	1.54	7.8	69.4–70.7	-	1.54	7.8	69.4–70.7	-	0.10	0.09
Tarsus length	qTAR1.1	1	0801_g7f8	**	1.99	18.8	13.7–16.9	-	-	-	-	-	0.55	0.60
	qTAR1.2	1	0196_69p16	***	1.97	11.8	67.5–71.7	-	2.94	41.2	67.5–82.3	75.6–82.0	0.62	0.57
	qTAR6	6	0810_65a23	***	3.38	49.0	7.37–12.4	-	-	-	-	-	0.64	0.58
	qTAR9	9	0553_18c8	****	2.09	19.2	46.6–55.3	48.6–53.3	3.47	15.4	51.1–52.3	51.1–52.3	0.63	0.57
	qTAR10	10	BTMS0129	*****	2.16	13.1	15.4–19.2	-	-	-	-	-	0.63	0.57
	qTAR15.1	15	BTMS0103	******	2.88	14.5	9.48–13.6	10.8–12.6	2.88	16.3	9.85–13.6	9.85–13.6	0.54	0.60
	qTAR15.2	15	0583_22I4	****	1.78	8.8	94.2–96.6	-	-	-	-	-	0.51	0.59
Leg length	qLEG6	6	0810_65a23	****	3.35	48.9	9.37–11.4	-	-	-	-	-	1.59	1.43
	qLEG15.1	15	BTMS0103	****	2.40	13.6	9.85–13.6	-	-	-	-	-	1.35	1.49
	qLEG15.2	15	0583_22I4	****	1.95	9.5	94.2–96.6	-	-	-	-	-	1.28	1.45
Eye length	qEYL1.1	1	0801_g7f8	**	2.43	19.5	0.0–18.9	12.7–14.9	3.89	14.3	10.4–10.4	10.4–10.4	2.46	2.61
	qEYL1.2	1	0360_2n11	***	2.97	31.7	27.7–31.7	27.7–30.7	-	-	-	-	2.40	2.57
	qEYL3	3	0795_67k24	****	2.00	10.4	42.2–62.6	-	-	-	-	-	2.69	2.54
	sEYL8	8	0869_70d5	**	2.30	16.6	65.0–87.2	-	-	-	-	-	2.47	2.60
	qEYL9	9	0553_18c8	*****	2.38	21.7	41.0–58.9	45.6–50.4	3.61	17.3	51.1–52.3	51.1–52.3	2.70	2.53
	qEYL15.1	15	BTMS0103	**	2.22	11.3	10.8–12.6	-	-	-	-	-	2.46	2.60
	qEYL15.2	15	0583_22I4	****	2.44	11.4	93.2–96.6	-	2.71	8.5	92.2–96.6	-	2.35	2.56
Eye width	qEYW1.1	1	0801_g7f8	*	2.06	16.5	0.0–18.9	13.7–15.9	-	-	-	-	1.04	1.10
	qEYW1.2	1	0360_2n11	**	2.92	31.4	27.7–33.7	27.7–32.7	-	-	-	-	1.02	1.08
	qEYW1.3	1	0196_69p16	******	2.81	16.0	60.5–77.3	62.5–75.6	4.00	32.3	61.5–82.3	62.4.5–81.0	1.13	1.06
	qEYW8	8	0869_70d5	***	2.75	18.8	63.0–91.6	-	-	-	-	-	1.04	1.10
	qEYW9	9	0152_56e6	**	2.28	19.3	38.0–51.1	44.6–47.6	3.86	24.9	35.0–49.6	36.0–49.6	1.04	1.10
	qEYW15.1	15	BTMS0103	****	2.54	12.3	9.85–13.6	11.6–12.6	-	-	-	-	1.04	1.10
	qEYW15.2	15	0583_22I4	******	2.04	9.6	91.2–96.6	-	-	-	-	-	0.99	1.08
Facet length	qFAC11	11	0930_40o1	****	2.14	11.3	70.9–77.1	-	1.99	9.7	70.9–71.9	-	0.02	0.02
	qFAC15	15	BTMS0103	****	1.86	9.7	10.8–13.6	11.6–11.6	-	-	-	-	0.02	0.02
Median Ocellus	qMOc1	1	0360_2n11	*	2.25	29.6	28.7–31.7	-	-	-	-	-	0.26	0.28
	qMOC2	2	0956_26c17	***	1.68	12.9	0.0–17.6	-	-	-	-	-	0.29	0.27
	qMOc5.1	5	0357_2o10	**	1.59	8.5	6.47–8.57	-	-	-	-	-	0.31	0.28
	qMOc5.2	5	0216_63a9	***	1.69	11.0	29.8–37.4	-	-	-	-	-	0.29	0.27
	qMOc6	6	0810_65a23	**	3.08	45.9	6.37–21.4	-	-	-	-	-	0.29	0.28
	qMOc7	7	0607_19k14	******	2.47	13.4	73.7–86.5	83.4–84.5	-	-	-	-	0.30	0.27
	sMOc8	8	0627_20n22	*	1.56	12.2	77.2–82.2	-	-	-	-	-	0.29	0.28
	qMOc9	9	0553_18c8	****	2.90	25.9	39.0–51.1	46.6–46.6	2.54	17.1	51.1–52.3	-	0.30	0.27
	qMOc12	12	0867_70k14	***	1.78	13.9	37.2–42.0	-	-	-	-	-	0.30	0.28
	qMOc13	13	BL16	****	1.91	13.7	0.0–19.1	10.1–12.7	-	-	-	-	0.30	0.27
	qMOc15.1	15	BTMS0103	***	1.82	8.5	11.6–11.6	-	-	-	-	-	0.26	0.28
	qMOc15.2	15	0583_22I4	******	1.84	9.0	96.2–96.6	-	1.77	6.7	96.6–96.6	-	0.25	0.28
Eye surface	qEYS1.1	1	0801_g7f8	**	2.24	19.0	0.0–17.9	-	3.54	11.7	10.4–10.4	10.4–10.4	2.04	2.26
	qEYS1.2	1	0360_2n11	**	3.19	34.4	27.7–32.7	27.7–31.7	-	-	-	-	1.94	2.20
	qEYS1.3	1	0196_69p16	**	2.24	13.3	64.5–72.7	-	-	-	-	-	2.35	2.11
	sEYS8	8	0627_20n22	**	2.63	18.7	60.0–91.6	61.0–91.6	-	-	-	-	2.37	2.20
	qEYS9	9	0553_18c8	****	2.53	22.6	38.0–57.9	45.6–49.6	3.52	13.5	51.1–52.3	51.1–52.3	2.40	2.14
	qEYS15.1	15	BTMS0103	****	2.48	12.5	10.8–13.6	-	-	-	-	-	2.02	2.25
	qEYS15.2	15	0583_22I4	*****	2.44	11.4	91.2–96.6	-	2.68	8.7	96.6–96.6	96.6–96.6	1.84	2.19
Ommatida number	qOMN1	1	0360_2n11	***	2.39	23.1	27.7–31.7	-	-	-	-	-	5132.62	5655.78
	qOMN3.1	3	0365_7n6	****	2.50	20.2	40.2–65.6	45.3–63.6	-	-	-	-	6077.68	5509.39
	qOMN3.2	3	0207_63e15	*****	2.15	13.2	88.2–96.4	89.2–96.4	2.62	21.0	78.5–87.3	80.5–87.3	5254.83	5772.37
	qOMN4	4	0304_9i13	****	2.71	35.7	63.1–80.7	64.7–80.7	-	-	-	-	6344.49	5559.78
	qOMN6.1	6	0810_65a23	****	2.41	26.4	20.4–30.4	-	-	-	-	-	6108.91	5517.56
	qOMN6.2	6	0725_82m14	****	2.39	26.1	73.4–87.8	-	-	-	-	-	5233.04	5767.28
	qOMN7	7	0338_2i5	***	1.75	16.5	113.4–132.2	-	-	-	-	-	6167.46	5545.42
	qOMN9	9	0553_18c8	*****	**3.42**	**24.6**	**44.6–67.4**	**47.6–64.4**	**2.48**	**16.7**	**52.3–53.3**	53.3–53.3	6142.32	5461.04
	qOMN12	12	0867_70k14	******	2.57	18.8	38.9–46.5	39.9–45.5	-	-	-	-	6243.95	5526.64
	qOMN13	13	BL16	****	2.18	19.6	0.0–13.8	0.0–11.7	-	-	-	-	6163.92	5522.71
	qOMN14	14	0655_82m17	****	2.12	10.7	52.0–56.9	-	-	-	-	-	5319.78	5803.45
	qOMN17	17	0608_19h1	*	1.10	8.4	46.4–57.6	-	-	-	-	-	5435.19	5773.70
CLS _ blue light	qBLU3	3	BT08	***	1.96	10.6	12.3–25.3	-	1.89	8.7	12.3–12.9	-	0.31	0.53
Dry weight	qDWE2	2	0956_26c17	**	1.74	18.3	20.6–23.5	-	-	-	-	-	0.24	0.20
	qDWE3	3	0795_67k24	****	2.27	18.0	39.2–56.0	40.2–53.0	-	-	-	-	0.26	0.20
	qDWE5	5	0357_2o10	*	1.50	9.2	4.47–8.57	-	-	-	-	-	0.27	0.21
	qDWE6	6	0810_65a23	*****	**3.28**	**26.1**	**17.4–34.0**	**24.4–33.0**	**3.58**	**18.4**	**27.4–31.7**	27.4–32.0	0.27	0.20
	qDWE9	9	0553_18c8	****	3.00	20.8	46.6–57.9	48.6–56.9	-	-	-	-	0.25	0.20
	qDWE10	10	BT20	******	2.07	14.5	103.6–126.5	-	2.47	8.1	116.0–116.0	-	0.18	0.23
	qDWE15	15	BTMS0103	****	3.05	14.1	9.85–13.6	10.8–11.6	3.87	14.2	9.85–14.6	10.8–13.6	0.18	0.23
Eye_PCA_1	qEPC1_1.1	1	0801_67f8	***	2.59	12.2	0.0–16.9	0.0–14.9	3.57	14.4	10.4–10.4	10.4–10.4	-0.75	0.36
	qEPC1_1.2	1	0360_2n11	*	1.68	9.2	26.8–27.7	-	-	-	-	-	-0.53	0.11
	qEPC1_7	7	BL05	**	1.48	7.3	152.7–157.4	-	-	-	-	-	-0.61	0.28
	qEPC1_9	9	0152_56e6	**	1.91	9.0	39.0–44.6	42.6–42.6	9.51	41.5	33.2–49.6	33.2–47.6	-0.82	0.26
Eye_PCA_2	qEPC2_6	6	0281_20d1	****	2.59	16.5	35.0–48.2	-	-	-	-	-	0.28	-0.17
	qEPC2_7	7	0338_2i5	**	2.03	21.6	105.1–146.2	114.4–117.9	1.86	18.5	136.1–141.1	-	-0.35	0.05
	qEPC2_12	12	0867_70k14	*****	3.68	18.9	34.4–46.5	36.2–45.5	3.06	22.7	39.9–44.5	41.1–44.5	-0.72	0.11
Size_PCA_1	qSPC1_6	6	0810_65a23	**	2.47	36.0	8.37–15.4	-	-	-	-	-	-1.27	0.25
	qSPC1_10	10	BTMS0129	***	1.62	7.5	18.2–18.2	-	-	-	-	-	-1.25	0.35
	qSPC1_15.1	15	BTMS0103	**	1.65	7.6	11.6–11.6	-	-	-	-	-	1.07	-0.34
	qSPC1_15.2	15	0583_22I4	**	1.74	8.6	94.2–96.5	-	1.74	8.6	96.5–96.5	-	1.92	0.08
Size_PCA_4	qSPC4_3	3	0795_67k24	**	2.06	10.6	41.2–55.0	-	1.80	7.6	42.2–48.2	-	-0.54	0.12
	qSPC4_15	15	0222_63d21	*******	2.39	10.8	36.9–49.8	-	13.4	47.2	36.9–68.7	42.2–68.7	0.70	-0.11
Size_PCA_5	qSPC5_13	13	0071_59g6	**	-	-	-	-	1.93	8.8	91.7–93.7	91.7–93.7	-0.26	0.10
	qSPC5_18	18	0187_69g1	**	1.23	8.3	27.0–51.0	-	4.60	34.8	45.0–46.0	45.0–46.0	-0.12	0.18

With the respective Kruskal-Wallis significance level and the closest marker useful for Marker Assisted Breeding. Significant QTLs at the genome wide level are indicated in bold.

^a^Kruskal Wallis significance levels: * = 0.1, ** = 0.05,*** = 0.01, **** = 0.005, ***** = 0.001, ****** = 0.0005 and ******* = 0.0001.

^b^LOD-scores higher than the LG specific 0.05% LOD-threshold indicates a significant QTL.

^c^The with composite interval mapping (IM) detected QTL interval under linkage group wide significant levels of *p* = 0.05 and *p* = 0.01.

^d^The with multiple QTL model mapping (MQM) detected QTL interval under linkage group wide significant levels of *p* = 0.05 and *p* = 0.01.

^e^The mean allelic value of allele ‘A' refers to the mean phenotypic value for the maternal allele (A or A’) and allele ‘B’ for the paternal allele, respectively.

When considering the 19 traits for which we found a suggestive or significant QTL with IM, 15 traits had at least 1 QTL with multiple QTL model mapping (MQM). Indeed, with the MQM mapping we identified 5 QTLs significant at the genome wide significance level of 0.05%, and 27 and 20 QTLs significant at the LG specific significance level of 0.05% and 0.01%, respectively (Table 3). These QTLs, distributed in 7 LGs, were explaining 6.7–41.2% of the phenotypic variation. For CLS under blue light conditions we found one suggestive QTL explaining 8.7% of the genotypic variation, while for CLS under UV light we found no significant QTL. For body mass of drones we found one significant (*qDWE6)* and two suggestive QTLs (*qDWE10* and *qDWE15*) while for the length of the radial cell we found one significant (*qRAC15*.*2)* and one suggestive QTL (*qRAC1*), cumulatively explaining 40.7% and 23.8% of the phenotypic variation, respectively. With MQM, we detected 1 to 3 suggestive QTLs for most of the eye traits: for the dorsal-ventral length (*qEYL1*.*1*, *qEYL9*, *qEYL15*.*2*), width (*qEYW1*.*3*, *qEYW9*) and total surface of the compound eye (*qEYS1*.*1*, *qEYS9*, *qEYS15*.*2*), the amount of ommatidia of a compound eye (*qOMN3*.*2*), the diameter of median ocellus (*qMOc9*, *qMOc15*.*2*), and the facet diameter (*qFAC11*) cumulatively explaining 40.1%, 57.2%, 33.9%, 21%, 23.8% and 9.7% of the phenotypic variation, respectively. For the amount of ommatidia of a compound eye we found also one significant QTL (*qOMN9*) explaining 16.7% of the phenotypic variation. For the different hind leg traits we found only suggestive QTLs for: (i) metatarsus length (*qMTL1*, *qMTL6*) explaining 34.2% of variation, (ii) tibia length and width (*qTIL15*.*2* and *qTIW6*, respectively) explaining 9.7% and 17.8% of variation, (iii) femur length and width (*qFML7*, *qFML15* and *qFMW11*, respectively) cumulatively explaining 28.8% and 9.3% of variation, (iv) trochanter width (*qTRW1*) explaining 7.8% of variation, and finally (v) three suggestive QTLs for tarsus length (*qTAR1*.*2*, *qTAR9*, *qTAR15*.*1*) explaining 72.9% of variation. Furthermore, we found one significant QTL with MQM for trochanter length (*qTRL6*) and metatarsus width (*qMTW6*) explaining 23.4% and 22.0% of variation, respectively.

### PC-QTL

**Fig 3:**
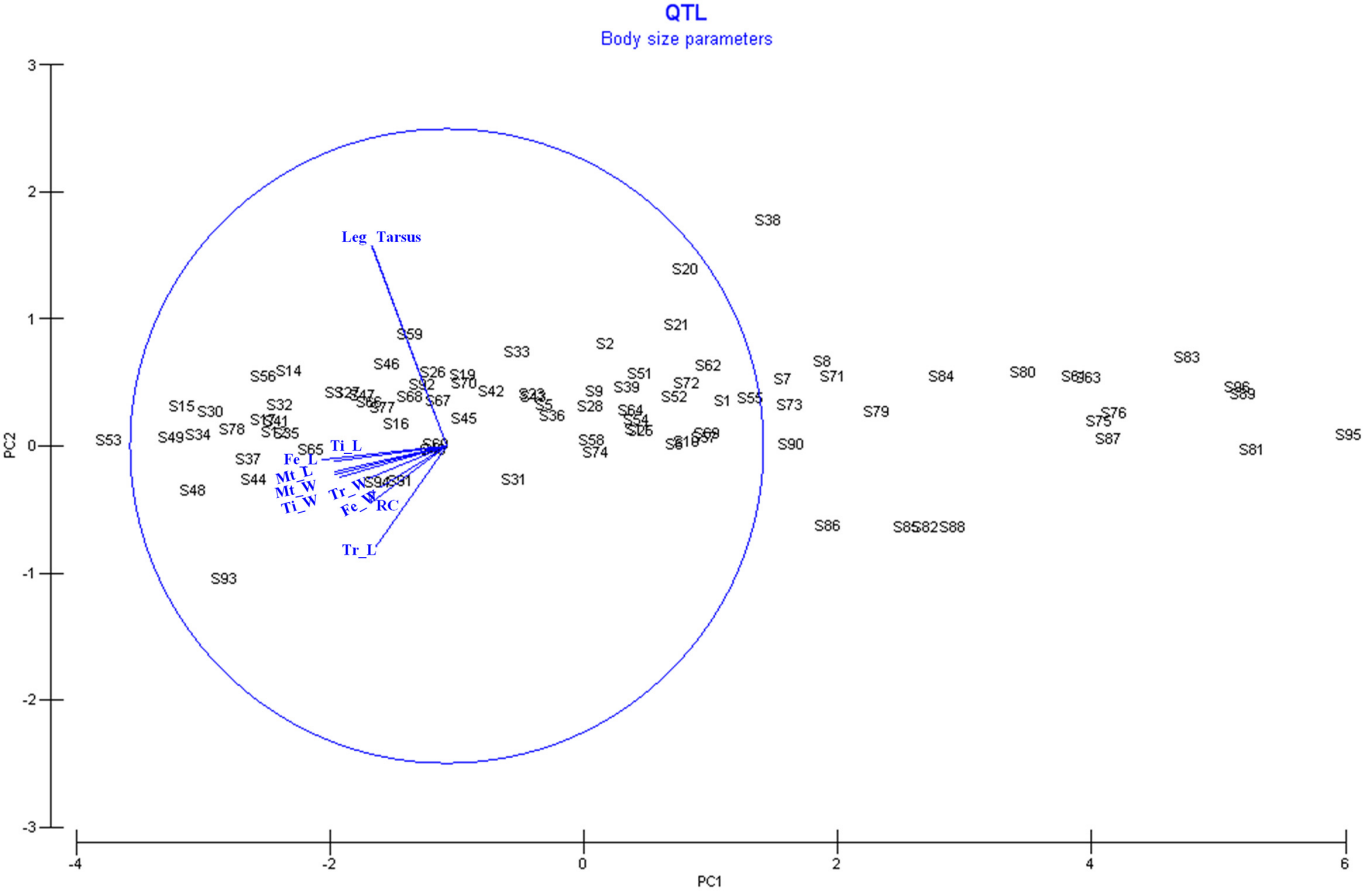
PCA graph of the different body size parameters.

The PCA for body size parameters showed 5 PCs of which two had eigenvalues higher than 1: 5.91 and 1.57 (PC1 and PC2, respectively; Fig 3, S4 Table). Together, these 5 PCs accounted for 89.5% of the total variance over these traits (S4 Table). In total, we found 8 suggestive QTLs for three PCs: PC1 (4), PC4 (2) and PC5 (2). The most informative PC is PC1 with 53.8% of the total variance of the trait while PC4 and PC5 accounted only for 6.5% and 6% of the total variance, respectively. Three of the four suggestive QTLs (*qSPC1_6*, *qSPC1_15*.*1* and *qSPC1_15*.*2*) of PC1 are linked with body size in general as confirmed by the QTLs of the individual body size traits (Table 3 and Fig 2). QTL *qSPC1_10* was only confirmed by the traits linked with tarsus size (Table 3 and Fig 2).

**Fig 4:**
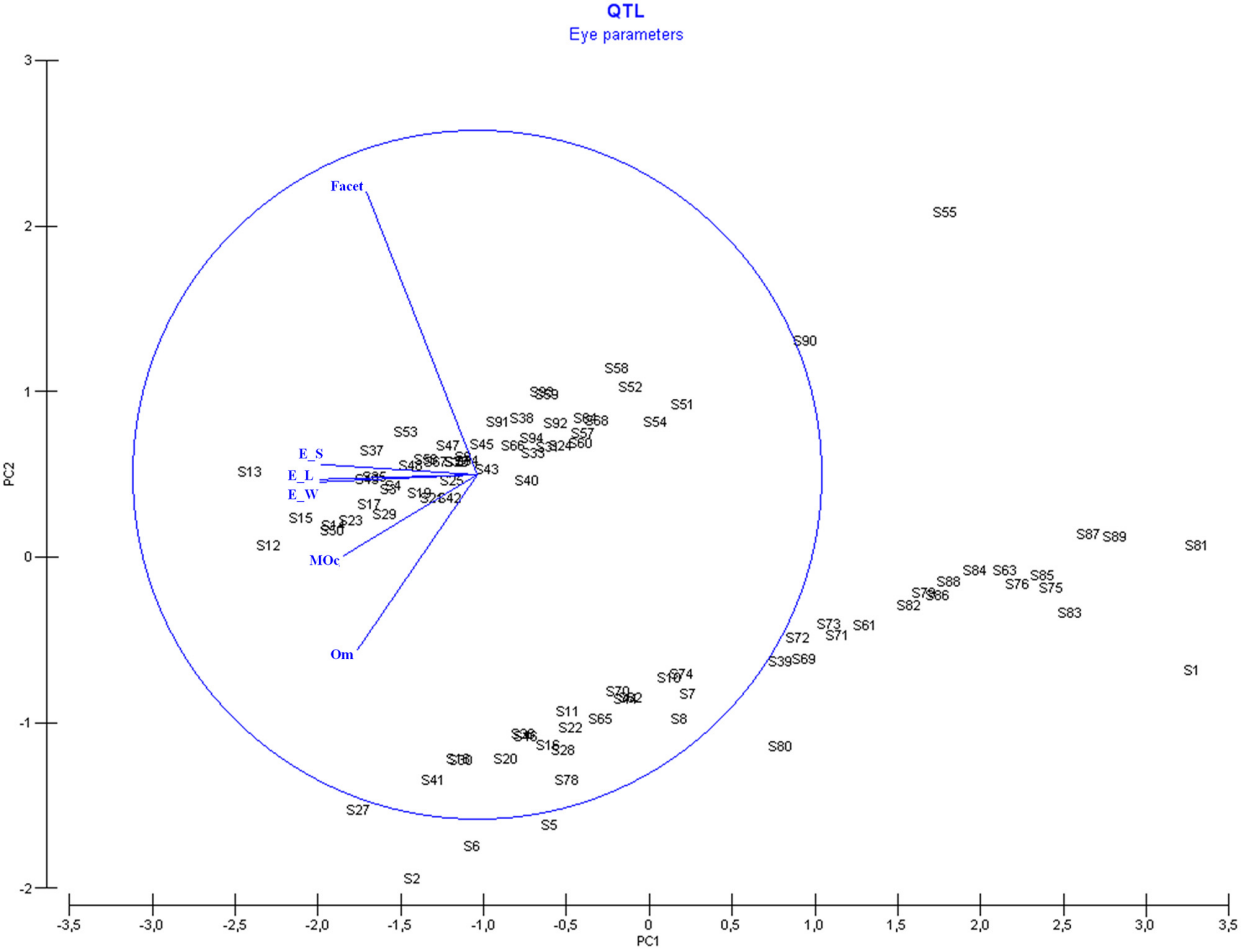
PCA graph of the different eye parameters.

The PCA on the different eye parameters showed 3 PCs which accounted for 74.1% (PC1), 10.9% (PC2) and 8.1% (PC3) of the total variance (Fig 4, S4 Table). Only PC1 had an eigenvalue higher than 1: 4.45 (S4 Table). All eye parameters showed negative correlations with PC1, ranging from -0.458 to -0.325. For compound eye length, eye width and eye surface, we found the highest correlations: -0.458, -0.456 and -0.453, respectively. Three of the 4 suggestive QTLs found for PC1 (*qEPC1_1*.*1*, *qEPC1_1*.*2* and *qEPC1_9*) were confirmed by the univariate suggestive QTLs for these three eye parameters, while QTL *qEPC1_7* was only confirmed by ommatidia number (Table 3 and Fig 2). The three suggestive QTLs for PC2 (*qEPC2_6*, *qEPC2_7* and *qEPC2_12*) correlated with the univariate QTLs found for median occelus and ommatida number on LG6, LG7 and LG12. (Table 3 and Fig 2).

### Epistatic interactions

We identified epistatic interactions using the program QTLMapper version 1.6 [46] (Table 4). One digenic interaction was identified for several body size parameters: leg size, tibia width, femur width, and trochanter length (Table 4). Furthermore, we found one epistatic interactions for the surface of the compound eye (Table 4).

**Table 4:** List of identified epistatic effects.

Trait	Ch-Ini^a^	Flanking markers	Ch-Inj^a^	Flanking markers	AA^b^
Ti_W	2–2	0428_13l21—BT05	17–1	0180_50k19–0321_15f5	0.006
Fe_W	2–9	0141_44j1–0940_33f14	3–1	0221_63h9—BT08	0.211
Tr_L	2–10	0940_33f14–0062_62f17	7–1	BT24–0526_4c10	0.008
Leg	5–2	0357_2o10–0216_63a9	14–11	0636_34m4–0466_5f11	0.096
E_S	5–7	0268_60h13–0535_15i17	15–1	BTMS0071—BT15	0.293

^a^ Ch-Ini and Ch-Inj represent the chromosome number-interval of the points being tested in the analysis.

^b^ AA is the epistatic interaction between both points (i and j).

### Candidate genes of light sensitivity

Candidate genes were identified for the suggestive QTL *qBLU3*. Therefore, we used SSR-marker BT08 which determine the QTL region, and the markers BT07 and 0291_60p14 as borders for the 95% C.I. of the QTL. The 64 genes around the markers BT07 and 0291_60p14 on the linkage group 3, were all identified as candidate genes (S5 Table). Based on the possible function in phototransduction and visual perception, locus Loc100650954, with as description a *Phosrestin-1-like* gene, was selected as the primary candidate gene.

## Discussion

Here, we have identified several suggestive and significant QTLs for morphological traits related to bumblebee light sensitivity, body mass, body size and several eye and hind leg traits (Table 3). The presence of multiple QTLs for 16 of the 20 traits clearly demonstrate their polygenic genetic character. For three traits: i.e. femur width, trochanter length and trochanter width, we identified only one QTL. We were unable to find a QTL for only light sensitivity under UV light conditions. As UV light is important for bumblebee foraging [47] and UV receptors are present in bumblebees [48], loci linked with UV detection could be under strong selection resulting in low genetic variation. Hence, it is quite possible that in our population with maximum 3 alleles for each locus, these loci could be present as homozygous. Furthermore, developmental and environmental factors could have caused no detection of QTLs for UV light. Finally, it is also possible that small effect QTLs are not detected here.

Our sample size (n = 92 to 96) was comparable or smaller in comparison with the sample sizes of other QTL studies in bumblebees, such as in Wilfert et al. [17–18] where sample size ranged from n = 76 to 359 and n = 153 to 173 respectively, depending on which trait and population was investigated. But our sample size was consistent with the sample size of other QTL studies, e.g. in plants (n = 90 or less; [49]). However, due to the Beavis effect, which causes biases in QTL effects, it is possible that small QTLs were not detected even with an increased sample size [50]. Thus only remarkably increasing the population size would increase the detection of yet unfound small effect QTLs. Although detection of all possible QTLs should be the ultimate target, the goal of this study was to identify genetic markers linked to some specific phenotypes for their later use in MAS. For this purpose, small effect QTLs are not as useful.

In this project, we found a suggestive QTL for light sensitivity under blue light conditions in a region where there is no QTL linked with body size or any other related morphological parameter. We already showed before that although larger bumblebees are better equipped to capture light, other genetic parameters influence bumblebee light sensitivity [12]. For this trait, we identified 64 candidate genes of which we identified the *Phosrestin-1-like* gene as the primary candidate gene due to the known phototransduction function of *Phosrestin-1* [51]. Indeed, in the Fruit fly (*Drosophila*) *Phosrestin-1*, also known as *Arrestin-B* or *Arrestin-2*, is identified as interacting directly with light-activated rhodopsin thereby activating the phosphorylation of metarhodopsin [51]. Furthermore, low and high levels of *Arrestin-2* in the rhabdomeres will enhance the photoreceptor sensitivity in weak light conditions, and prevent hyperactivity of the photoreceptors in strong light conditions [51]. Further research is necessary to validate this gene’s impact on improved light sensitivity in bumblebees and its effect on foraging activity in diminished light conditions.

Not surprisingly we also found several overlapping univariate suggestive and significant QTLs between the length of the radial cell, as measurement of bumblebee body size, and most of the other measured size related morphological parameters (Table 3 and Fig 2). Several QTLs overlapped also between drone body mass and body size: e.g. one QTL region at LG6, LG9 and LG15, but a more interesting result was that not all QTLs overlapped for these parameters (Table 3 and Fig 2). Indeed, drone body mass showed unique QTL regions at LG2 (*qDWE2*), LG3 (*qDWE3*), LG5 (*qDWE5*), and LG 10 (*qDWE10*), while radial cell and body size parameters had unique QTL regions at LG1, LG7 and LG15. These regions were confirmed by the PC-QTL. Indeed, PCs showed size related suggestive QTLs on LG6, LG10 and LG15. Only one suggestive QTL on PC4 overlapped with one of the unique univariate body mass suggestive QTLs on LG3 (Fig 2). The presence of these specific genetic regions for drone body mass and body size indicates regulation of different genes.

In this study, we found only a few epistatic interactions for some traits in our *B*. *terrestris* population. This result is comparable with the results of Wilfert et al. [17]. In the latter study, the authors found only minor epistatic interactions for the ‘household’ trait: body size and more epistatic effects for fitness-relevant traits such as the general immune defense of encapsulation and the susceptibility to infection by *C*. *bombi*.

Although preliminary, these results support the idea of marker assisted breeding towards larger bumblebees, with the use of the identified markers at those unique QTLs. However, before these QTLs could be used they need to be validated in a broader genetic background, using multiple bumblebee populations. For QTL studies it is common that most of the QTLs found in one population will not withstand this validation, even if there are only very small differences in the experimental setup [17]. Indeed, in Wilfert et al. [17] the authors used three bumblebee populations in which they detected several QTLs for the traits: *Crithidia* infection intensity, general immune response (encapsulation of a novel antigen), and body size (measured by the length of the radial cell of the forewing) at different places and on different linkage groups. Wilfert et al. [17] found 10 QTLs for body size measured as the size of the radial cell of the forewing, with only low phenotypic effects (between 2% and 15%). Of those 10 QTLs, only one QTL (*BS-8*) was recovered in our study (*qRAC15*.*1*). This suggestive QTL, which accounts in our study only for 9.6% of the phenotypic variation, is a potential candidate for use as a genetic marker in MAS. Thus, in our study we were not only able to confirm a minor QTL for body size from Wilfert et al. [17], but we also found several significant and suggestive QTLs explaining more than 15% to even 50% of the phenotypic variation within a certain trait which are restricted to our bumblebee population and need validation in a broader genetic background.

In conclusion, our study identified one suggestive QTL for light sensitivity under blue light conditions explaining 10.6% of the phenotypic variation of the trait. Furthermore, we identified a list of 64 possible candidate genes for this trait of which the *Phosrestin-1-like* gene is identified as the primary candidate gene. Finally, we also found several significant and suggestive QTLs for body weight, body size and the morphological parameters of the eye and hind leg. Further research needs to determine if the QTLs found here, resist validation in a broader genetic background and if some of the SSR markers linked with those QTLs could be used as genetic markers in marker assisted breeding, to improve the pollination service of bumblebees.

## Supporting Information

S1 Fig Histogram of all investigated morphological traits. (PDF)

S1 Table Characteristics of the microsatellite markers used. From each SSR marker we present the forward and reverse primer sequences, GenBank accession number, annealing temperature (*Ta*), the observed size range of the PCR product, the location (LG) and the original reference.
(PDF)

S2 Table Distribution information of the 100 markers used for preliminary linkage mapping. The number of markers on each linkage group (n), the size of this linkage group (size LG), and the minimum (Min. d) and maximum (Max. d) distances between two markers on each linkage group.
(PDF)

S3 Table Kolmogorov-Smirnov test of normality for each trait. (PDF)

S4 Table PCA of the different body size traits and eye parameters. The eigenvalues and eigenvectors of the PCA are given for the different body size traits and the eye parameters.
(PDF)

S5 Table List of candidate genes for critical light sensitivity of bumblebee drones in blue light. List of the place, accession number, name and annotation information of all genes, at suggestive QTL qBLU3 on LG 3, which can all be linked with the critical light sensitivity of bumblebee drones in blue light.
(PDF)
