# Supplementary material for: Quantitative Trait Loci for Light Sensitivity, Body Weight, Body Size, and Morphological Eye Parameters in the Bumblebee, Bombus terrestris
Source: PLoS One. 2015 Apr 30;10(4):e0125011. doi: 10.1371/journal.pone.0125011 (PMC4415782; doi:10.1371/journal.pone.0125011)
Supplement: S1 Fig — (PDF) [file pone.0125011.s001.pdf]

Quantitative trait loci for light sensitivity, body weight, body size, and morphological eye parameters in the bumblebee, *Bombus terrestris*

Kevin Maebe[1], Ivan Meeus[1], Jan De Riek[2], Guy Smagghe[1,*]

S4_Fig: Histogram of all investigated morphological traits. Forewing radial cell length (RC), metatarsus length (MT_L), metatarsus width (MT_W), tibia length (Ti_L), tibia width (Ti_W), Femur length (Fm_L), femur width (Fm_W), trochanter length (Tr_L), trochanter width (Tr_W), tarsus length (tarsus), length of hind leg (Leg), length of compound eye (E_L), width of compound eye (E_W), total surface of compound eye (E_S), diameter of facet (Facet), total numbers of ommatidia (Om), diameter of median ocellus (MOc), body mass (weight), and the transformed critical light sensitivity in blue and UV light conditions (log_blue and log_UV, respectively).

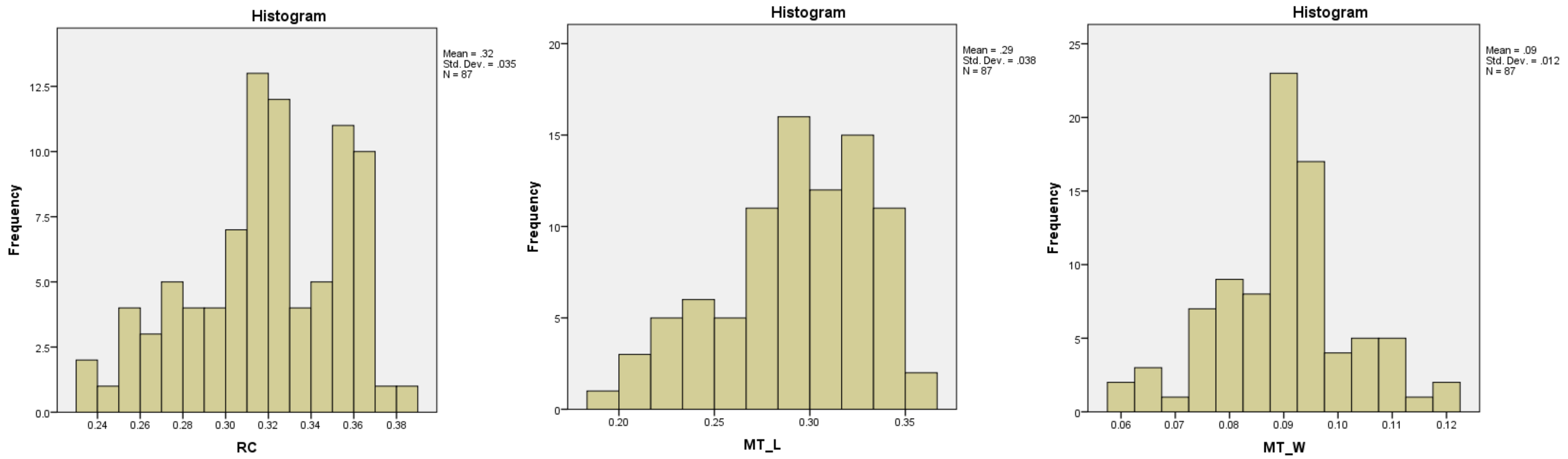

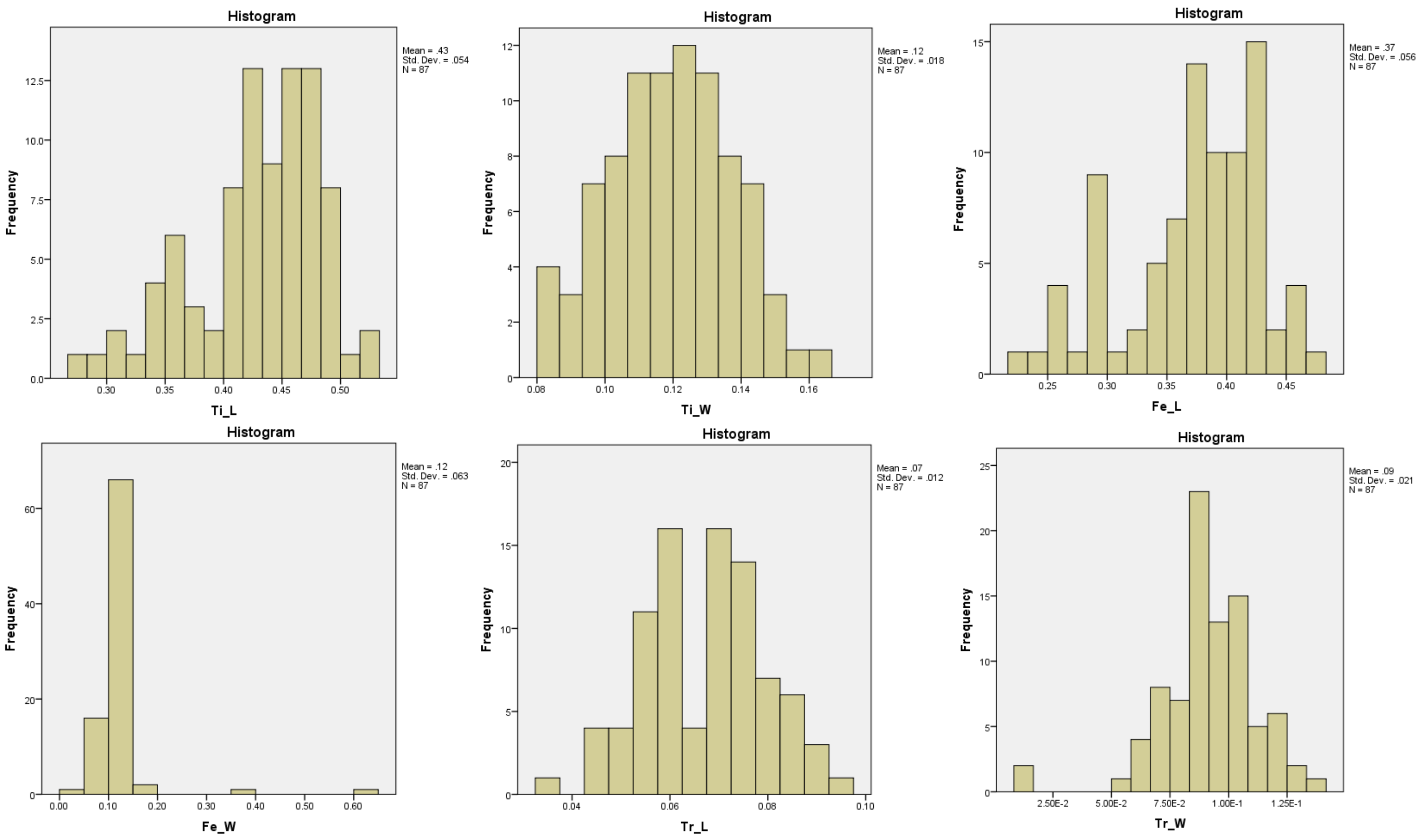

Histogram
Mean = .43
Std. Dev. = .054
N = 87
Frequency
Ti_L
Histogram
Mean = .12
Std. Dev. = .018
N = 87
Frequency
Ti_W
Histogram
Mean = .37
Std. Dev. = .056
N = 87
Frequency
Fe_L
Histogram
Mean = .12
Std. Dev. = .063
N = 87
Frequency
Fe_W
Histogram
Mean = .07
Std. Dev. = .012
N = 87
Frequency
Tr_L
Histogram
Mean = .09
Std. Dev. = .021
N = 87
Frequency
Tr_W

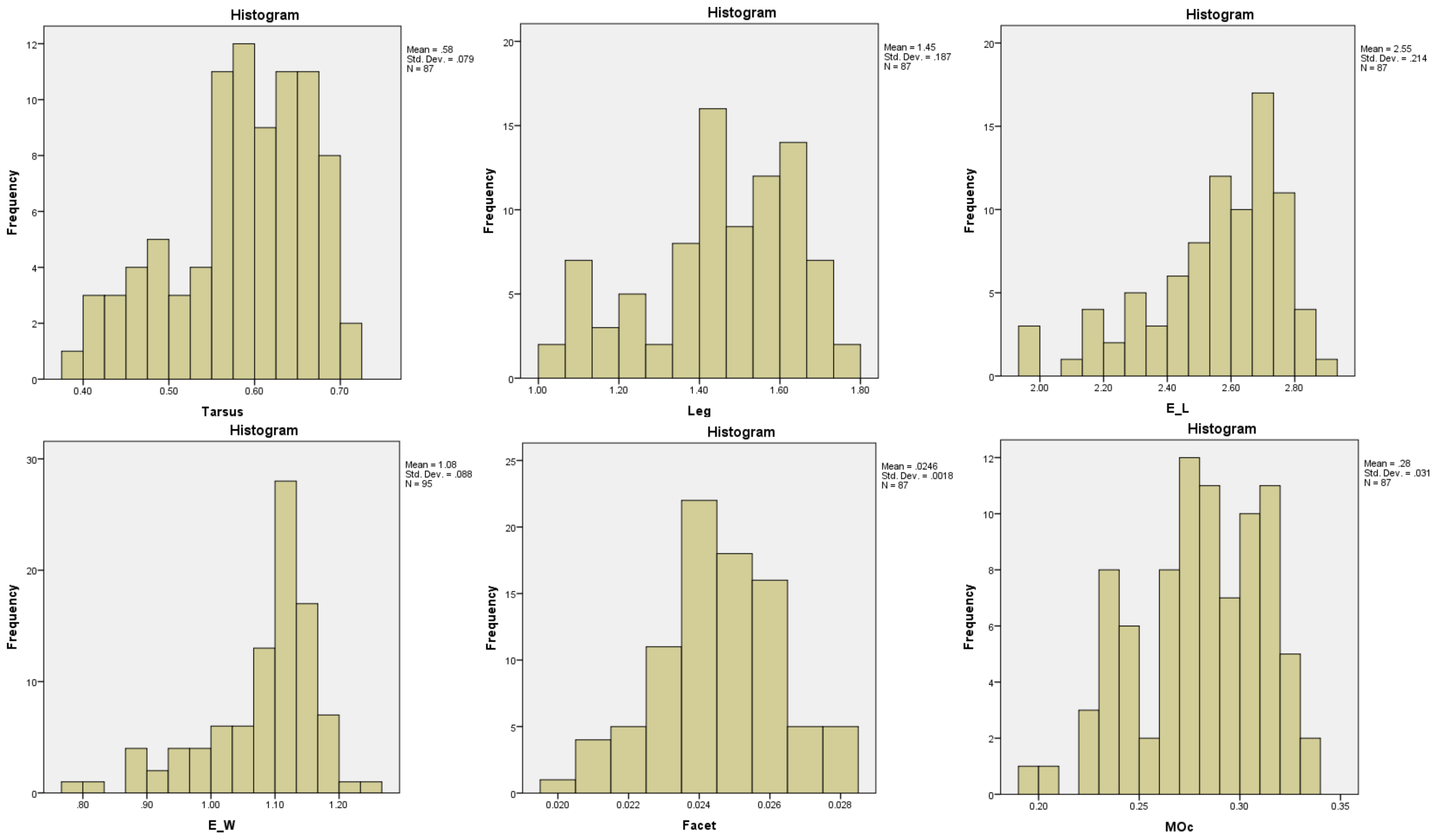
Histogram
Mean = .58
Std. Dev. = .079
N = 87
Frequency
Tarsus
Histogram
Mean = 1.45
Std. Dev. = .187
N = 87
Frequency
Leg
Histogram
Mean = 2.55
Std. Dev. = .214
N = 87
Frequency
E_L
Histogram
Mean = 1.08
Std. Dev. = .088
N = 95
Frequency
E_W
Histogram
Mean = .0246
Std. Dev. = .0018
N = 87
Frequency
Facet
Histogram
Mean = .28
Std. Dev. = .031
N = 87
Frequency
MOc

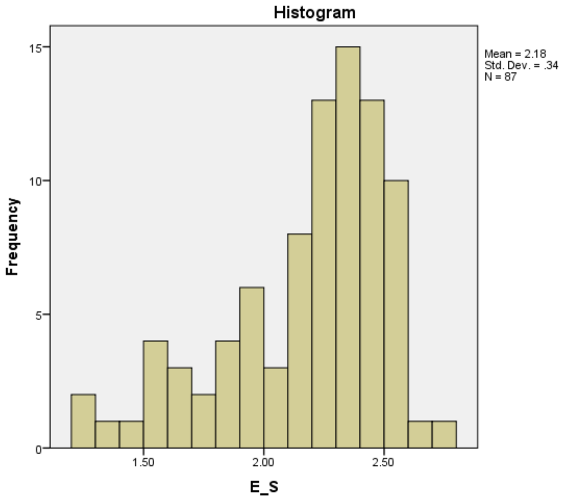
Histogram
Frequency
0
5
10
15
1.50
2.00
2.50
E_S
Mean = 2.18
Std. Dev. = .34
N = 87

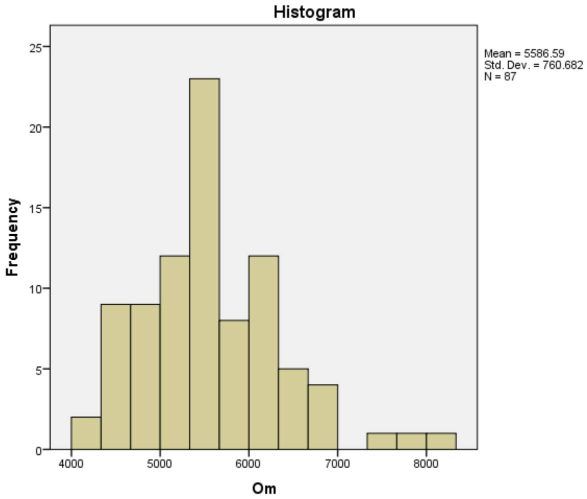
Histogram
Frequency
0
5
10
15
20
25
4000
5000
6000
7000
8000
Om
Mean = 5586.59
Std. Dev. = 760.682
N = 87

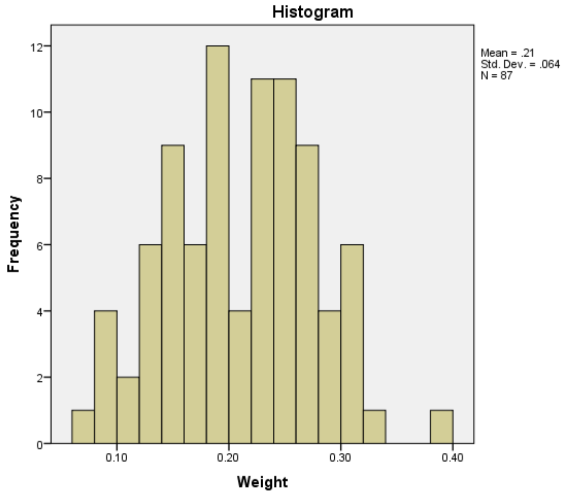
Histogram
Frequency
0
2
4
6
8
10
12
0.10
0.20
0.30
0.40
Weight
Mean = .21
Std. Dev. = .064
N = 87

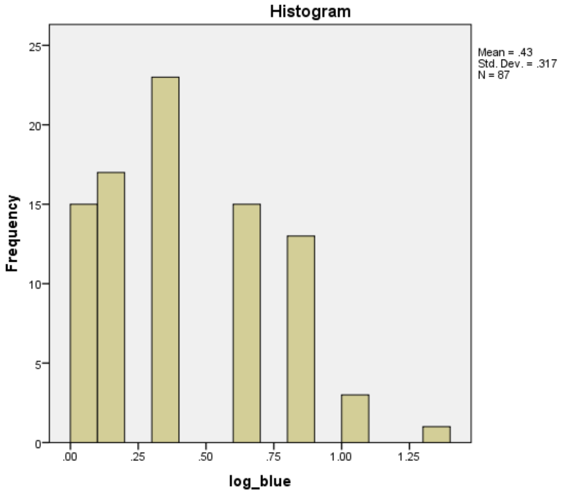
Histogram
Frequency
0
5
10
15
20
25
.00
.25
.50
.75
1.00
1.25
log_blue
Mean = .43
Std. Dev. = .317
N = 87

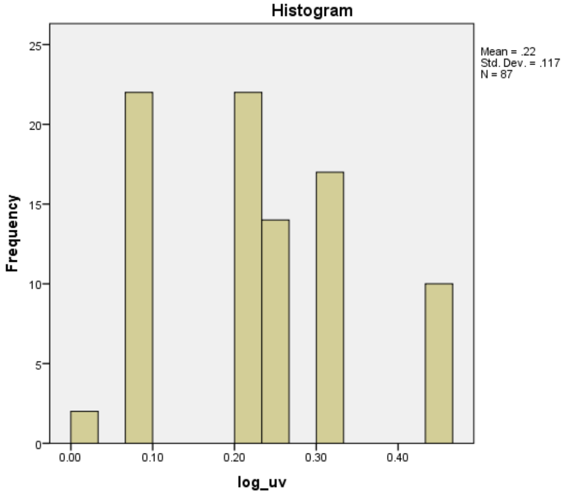
Histogram
Frequency
0
5
10
15
20
25
0.00
0.10
0.20
0.30
0.40
log_uv
Mean = .22
Std. Dev. = .117
N = 87
