# Supplementary material for: Quantitative Trait Loci for Light Sensitivity, Body Weight, Body Size, and Morphological Eye Parameters in the Bumblebee, Bombus terrestris
Source: PLoS One. 2015 Apr 30;10(4):e0125011. doi: 10.1371/journal.pone.0125011 (PMC4415782; doi:10.1371/journal.pone.0125011)
Supplement: S1 Table — From each SSR marker we present the forward and reverse primer sequences, GenBank accession number, annealing temperature (Ta), the observed size range of the PCR product, the location (LG) and the original reference. (PDF) [file pone.0125011.s002.pdf]

Quantitative trait loci for light sensitivity, body weight, body size, and morphological eye parameters in the bumblebee, *Bombus terrestris*

Kevin Maebe[1], Ivan Meeus[1], Jan De Riek[2], Guy Smagghe[1,*]

S1_Table: Characteristics of the microsatellite markers used. From each SSR marker we present the forward and reverse primer sequences, GenBank accession number, annealing temperature (*Ta*), the observed size range of the PCR product, the location (LG) and the original reference.

Marker	Accession number (GenBank)	LG *B.t.*	Forward primer sequence	Reverse primer sequence	*Ta*	Range	Source
0007_47n22	FQ377670	B11	GGTTATGATTGCACACTGTTT	GCACATTAAATTATTGCGTACA	60	152-158	Stolle et al., 2011
0043_71h15	FQ377672	B03	AATTTATGCGAAGATGATGTTA	TTAGTAACTGACTGCTGCTACG	60	164	Stolle et al., 2011
0053_71f10	FQ377673	B02	GTAAAGCGGAGAAACAAGATAG	CTTCTTCCACCTCTCATTTG	60	168	Stolle et al., 2011
0062_62f17	FQ377675	B02	TAAAATTGCTGGCTGGAAGCAT	CAGAGAACAACATCGTGGCAAA	60	197-213	Stolle et al., 2011
0071_59g6	FQ377677	B13	TACGATTCACCGATCTTAAATA	TTAATCGGAAGACACTGGAC	60	187-191	Stolle et al., 2011
0078_59o4	FQ377680	B14	AATCTCGTAATTCCAAGCTTAC	GATTTACTTGGGCAGACTTTAG	60	150-156	Stolle et al., 2011
0083_47g5	FQ377681	B13	TCTTAATCGATTCAAACATCCT	GATGAGTGTCATCCTTCTGAAT	60	136-148	Stolle et al., 2011
0103_38m23	FQ377683	B05	GTATCGCGATTGGTAATTATG	ACATCTTTGTATCTTCGAATCC	60	187	Stolle et al., 2011
0141_44j1	FQ377689	B02	CTAGGCCAGAATAGAGTCGTC	AGATTCGAGTGCTTTCCTCT	60	137-157	Stolle et al., 2011
0152_56e6	FQ377691	B09	GAACCTGTGTTCCTCTCGTA	TCTACTACACTTTGTCCGTTGA	60	146-148	Stolle et al., 2011
0162_69a8	FQ377693	B12	GAAGGAGTTGAATCATTAGGTC	TTCGTAGGGTGATAGAGGTG	60	155-157	Stolle et al., 2011
0172_44e21	FQ377695	B04	ATAATGCAGTTCCTCGAGTCT	GCTGTATTGGGTAGAAGAAAGA	60	148-150	Stolle et al., 2011
0177_44p18	FQ377696	B03	TTGACGATATTCTCTCACGATA	GCGTTTCTATCAGAAGCTACAC	60	169-172	Stolle et al., 2011
0180_50k19	FQ377697	B17	CCTTCCTGGAGGTAACCTTCTT	TTCATACGCGAGGTATGTGGAG	60	216	Stolle et al., 2011
0187_69g1	FQ377698	B18	TCTTGTATTAACCCAACGTACA	GCAGCTAACGGATCTTATTCTA	60	161-169	Stolle et al., 2011
0195_69j13	FQ377699	B01	CTGAACAATAATTACCGACAGA	GACAATTTCGATTACGAGACTT	60	150-154	Stolle et al., 2011
0196_69p16	FQ377700	B01	CGCTGAATCTAGACGCTATAA	ATCAGTGGCAATACATGTAAAC	60	188-194	Stolle et al., 2011
0198_69f24	FQ377701	B01	AAATAGCTCGACACTGAGAGAC	ATCCATAAGCGTGTAAGAAAGT	60	164-168	Stolle et al., 2011
0207_63e15	FQ377704	B03	TGTCTTTACGTCCATGTTACAC	CGTTCTCTATATACGGCAAGTT	60	193-197	Stolle et al., 2011
0216_63a9	FQ377705	B05	TCATAACGTTTCACATCTTGAC	GTCTAAAGTTCTATGCCACGTT	55	175-177	Stolle et al., 2011

Marker	Accession number (GenBank)	LG *B.t.*	Forward primer sequence	Reverse primer sequence	*Ta*	Range	Source
0221_63h9	FQ377706	B03	GTTATCGTATTTACACCGGAAC	TTTCTTCGCAAGATAGAGAGAG	55	154-158	Stolle et al., 2011
0222_63d21	FQ377707	B15	TCAATCTTCGATCTACGTAACA	AAATACGTGGCATTAACTCG	60	165-169	Stolle et al., 2011
0232_81d20	FQ377710	B13	GCGAGTCTGTACAATGAATATG	ACGGAACAACGAACAACTTA	60	171-177	Stolle et al., 2011
0242_81d21	FQ377711	B09	CCTCGATATCACCATAGGAA	ACAGATGTATCCGTGCAGTT	60	183-187	Stolle et al., 2011
0244_81l8	FQ377712	B13	AGAAGCTACAGTTGATGAGGAT	GAGTTCTCTGCTTGTCTGATG	60	142-150	Stolle et al., 2011
0255_16m20	FQ377713	B16	TCGTAAAGCTGAGAGCTATAAA	AAGATCGAGAGAACAGGATTAG	60	155-161	Stolle et al., 2011
0266_60m24	FQ377714	B10	AACGTTTCGAGAATAATAAAGC	AGGTAAAATGAACGAGACAAAG	60	213	Stolle et al., 2011
0268_60h13	FQ377715	B05	TCTGTAAACACAAGAGAAGTTCA	TCTGACGGTAGGACAATACTAA	60	210-203	Stolle et al., 2011
0275_90o12	FQ377716	B04	AGGACATCTGGTCTTAATGAAA	TACGCAGATCGTTGTTATACAT	60	163-165	Stolle et al., 2011
0281_20d1	FQ377719	B06	GTAGCCTTCTCTATGCCATTT	AGAACGGGTACGTGTAAATAGT	60	131-135	Stolle et al., 2011
0289_60l4	FQ377722	B10	TGTTGTATTGTGAATGTACACG	GCATGTAAACCAGACTTAATTG	54	162-164	Stolle et al., 2011
0291_60p14	FQ377723	B03	TCTGCTACGTTTAATTACTGGA	ACCCTCTTACCTATTTGGTGTA	54	196	Stolle et al., 2011
0292_60b14	FQ377724	B03	GCGTACGATATAAGGAAAGAGA	GTGAGTTCGAGCAATAATCC	55	153-167	Stolle et al., 2011
0294_10o4	FQ377725	B09	AGTACGATAAAGCCAGGAAAG	TGTATGCCTATTGTACGAGTGT	55	169-177	Stolle et al., 2011
0303_10b14	FQ377727	B02	AAAGTGTCATCGACCAGAAG	CTCGTTCGTTTAATTAGTCGTC	60	164	Stolle et al., 2011
0304_9i13	FQ377728	B04	GTATGAGTGAGTGATGTGCAAG	CCCTTCATCTCTGAACAATATC	55	154-160	Stolle et al., 2011
0320_15c24	FQ377729	B12	TTTCATTGTTTCCCTATTTCA	CCTTACGTACTTCCGTTATCTC	55	162	Stolle et al., 2011
0321_15f5	FQ377730	B17	ATGACAAAATATAGCACTGTATGTGT	GCAGAACGAACAAGATGTTCAA	60	210-212	Stolle et al., 2011
0336_1l24	FQ377734	B13	ACTTAGACACGCCTCAATTATC	GTTGAACCTTTGTTGAGAAGAT	60	114-136	Stolle et al., 2011
0338_2i5	FQ377735	B07	TCGTACTTCGTTCATCTAATCA	GGAATTTGTAATTTCGTTTGTT	54	172-174	Stolle et al., 2011
0357_2o10	FQ377736	B05	CGACAGTTGTTATTACGATGAA	CCCTTTAAGCAGACGTATTTAG	60	163-171	Stolle et al., 2011
0360_2n11	FQ377737	B01	ATAATTCCCGAACGAATGTC	TAACGTTATCCGGGTACAAA	55	231-237	Stolle et al., 2011
0365_7n6	FQ377738	B03	GCCATCAATAGATCAAAGAAAT	CTCCTCGGTCTGTTGTTTAT	60	138-140	Stolle et al., 2011
0370_12o14	FQ377739	B11	GGAACACGTATTACACAAAGTCT	CGAGAGACAGAGAGAAAGAAAG	54	221-225	Stolle et al., 2011
0374_12n22	FQ377741	B09	CCAGAGTGAGAAAGAGAGAGAG	CATAAATGTCCCACCTACATC	60	133	Stolle et al., 2011
0379_5i10	FQ377743	B07	AGAGAGAAAATCGAGGAAAAG	CGCAAAGTATTGCATAAATAAG	54	201-205	Stolle et al., 2011
0382_5e22	FQ377744	B11	GATAGAAACGACCAGGCTTAT	GGAACGAGTAACAAGGTAGAGA	60	167-169	Stolle et al., 2011
0392_16e2	FQ377746	B09	AATTATCATCGCGTAGGTACA	GTAAGCGTTTACACGAACAAC	60	183	Stolle et al., 2011

Marker	Accession number (GenBank)	LG *B.t.*	Forward primer sequence	Reverse primer sequence	*Ta*	Range	Source
0403_13g6	FQ377748	B07	ATTTACTGCTCGATACTTTCGT	ATCGAGTTCTTATTTCATCCTG	60	175-179	Stolle et al., 2011
0428_13l21	FQ377750	B02	AAGGTAACAGAAGAGACGATTG	GTCATTGTCAAGAGGTGGAG	60	154-156	Stolle et al., 2011
0466_5f11	FQ377753	B14	GTGTGTGCAAATAGCTACAGAT	GTCCCTTTACCCTTTAGATACC	60	163-171	Stolle et al., 2011
0482_3k3	FQ377756	B13	ATGGCAAGTGTTCTCGTACT	ATCTATTTACCAGCGAAGCTC	54	204	Stolle et al., 2011
0487_3f11	FQ377758	B12	CACCTTACAATATAGGTCAGTTGT	GTCTAGGTGCTCAATGGATATT	55	144-152	Stolle et al., 2011
0503_3m14	FQ377760	B13	ACAACTAATTTGCTGCCTCTAC	TAGGATCATTAAACGAGTCTCC	60	167-183	Stolle et al., 2011
0526_4c10	FQ377761	B07	TCACGTTGTGTCAACTGTAAA	AGATTCAAGACGAAAGAATTTG	55	162-176	Stolle et al., 2011
0533_15c9	FQ377762	B07	CGAAGAACATAAGCAGAGGTAG	CTTCCTCTTCGGTTCTCATAC	60	160	Stolle et al., 2011
0535_15i17	FQ377763	B05	GTCGCATTAAATACAAGCTACA	TTTCAAAGTGATATACAGGGAAG	55	139-157	Stolle et al., 2011
0543_6o7	FQ377764	B12	AGCTAAATTAACCAACACCAAT	GGCAGAGGAATATGATACAAGT	55	163-181	Stolle et al., 2011
0553_18c8	FQ377768	B09	AGGATTCCATTTCGAGAATAA	CAATGCACTACAAAGTTAGTTCC	54	236-242	Stolle et al., 2011
0566_20o5	FQ377772	B03	TGTTAATCGTCTGTCACCTTT	GTAGCAAGAAGTAGGCAAATG	55	180	Stolle et al., 2011
0576_20n23	FQ377773	B08	CCGTGCTATACTCACATTTCTA	ACGATCTATGTACCACGATTCT	55	166-168	Stolle et al., 2011
0579_22m16	FQ377774	B10	GCCAGGTACATATATCCCTATT	TTCCATATTTGCTGTCACTTT	60	186-202	Stolle et al., 2011
0583_22l4	FQ377776	B15	CGAATGAAATTAGCTCCACTAC	CAATTTCTTTCTCTTACGAAGC	60	126-140	Stolle et al., 2011
0594_19n18	FQ377778	B15	TTCAGAAGCATTCTCGAATTA	ATACGAAGAGAAATAGGGTACG	60	208-212	Stolle et al., 2011
0606_19m4	FQ377781	B02	ATAACGAGGAGAGTGGTAACTG	GTCTCCTAGCATCTTCTTTGTAA	60	235	Stolle et al., 2011
0607_19k14	FQ377782	B07	TCCATATGAAGATCACAGAGAA	TTAATCAGTGCATGCTTAGTGT	60	154-160	Stolle et al., 2011
0608_19h1	FQ377783	B17	GATCGATAAACGTCCAACTTAC	ATGGATTCTATCATCAATTCGT	60	209-211	Stolle et al., 2011
0613_19h23	FQ377784	B12	TTTATTCTACGCAAATGGTG	TATCAATATCAGTATCGGCATC	60	190-222	Stolle et al., 2011
0614_19d6	FQ377785	B08	AAGTAGAACGGATACAGAAACG	ACTCCAGTATGAGATGGAAGTC	60	186-196	Stolle et al., 2011
0627_20n22	FQ377789	B08	CGTGTAAACACACATAAAGAGC	GTTTCGTCTTCGCTCTAGATAC	60	176-192	Stolle et al., 2011
0631_34k4	FQ377790	B05	ATAACCGAAAGACAAAGTTCAC	GCTCTTGCTCTTTCTTTATCTT	60	160	Stolle et al., 2011
0632_34i8	FQ377791	B03	TTCCCGTATTATGTAACTCAGA	GCTTGGAGAAGATAGTTAAACG	60	189	Stolle et al., 2011
0636_34m4	FQ377792	B14	AGTGAAAGTTGACGAAGAACA	CCGAGATCTCTCTCTGTACTGT	60	145-151	Stolle et al., 2011
0644_83i19	FQ377796	B06	CATTGTCGAGTGAATATCGAG	TAGAAATCATTGCAACAGAGAA	60	166-168	Stolle et al., 2011
0646_83e8	FQ377798	B04	GTTTCTCTCTTCCCTCTTTCC	AAGATGCAGAGGAAAGTAAATG	60	155-165	Stolle et al., 2011
0655_82m17	FQ377800	B14	TACATCTACTCGTCTCCCTCTC	ACGGATAGACAAACAGAGAATC	60	134-136	Stolle et al., 2011

Marker	Accession number (GenBank)	LG *B.t.*	Forward primer sequence	Reverse primer sequence	*Ta*	Range	Source
0669_84l15	FQ377804	B09	TGCTTGACGAATATGAAATG	AAACAGATCGAGAGAAAGAGAG	60	170-180	Stolle et al., 2011
0686_86i9	FQ377807	B14	AAAGATAGAAGAAGGAAGCACA	TCGAACTATCCACCAGCTATAC	60	130-136	Stolle et al., 2011
0712_84o11	FQ377811	B06	TTTCGATGGTGGTTTGTACT	CTTGCCGATATATTACCTCTTC	60	198-210	Stolle et al., 2011
0725_82m14	FQ377814	B06	TCTATCAAACACGTAAAGCGTA	ATTTATGACCTCTCCTCTCACA	60	161-195	Stolle et al., 2011
0731_75c7	FQ377815	B09	GTGTACAGGCATACAGAAAGTG	GAAAGAGGAAGAGAGAAATCAA	60	181-185	Stolle et al., 2011
0742_75d18	FQ377818	B05	ACAAAGTGGTGCCATATTTATT	AGATACTGTGACCTAAGGGAAA	60	170-174	Stolle et al., 2011
0745_75i17	FQ377819	B13	GAATACACATGTCTCTGGTTCA	GAGAGTCGATCTTGTGAGAGAT	60	168	Stolle et al., 2011
0751_11c2	FQ377820	B16	CTTCAACGTCCATCCTGTATC	CCATCTTTTTCACCCTTGTATATAC	60	193-199	Stolle et al., 2011
0752_11m6	FQ377821	B10	AAATTAACACCAGCGGTTCT	AACTTTCTAAGCGTGTGCAG	60	163-175	Stolle et al., 2011
0774_1a20	FQ377826	B14	CTCCCTCTCTCTCTTTCTCTTT	CCCATATCAGTGACAAAGAAC	54	175-189	Stolle et al., 2011
0777_1j15	FQ377827	B10	TGCACTCATAGAATGAGAAAGA	GGATTTGTGGACGTTAATTG	55	152-166	Stolle et al., 2011
0795_67k24	FQ377830	B03	GAATTCCCAGAGAACAATTTC	TAAATTTACGAGTTTGCACAAG	60	161-169	Stolle et al., 2011
0801_67f8	FQ377832	B01	ATACTGTACGCGCATGTAATAA	TAATTTCTTCTCCTCGTTTCTC	55	167-195	Stolle et al., 2011
0803_67i16	FQ377833	B10	CCAGGTAAAGGTAACAAATCAC	GTGTTAGGGACACGTCAAGT	60	172	Stolle et al., 2011
0810_65a23	FQ377835	B06	TTAACAAATCCGAATTTAAAGG	GATAGTGGTTGCTTGTCATCTT	55	136-140	Stolle et al., 2011
0811_65m2	FQ377836	B08	TACAACTTGACGAGGAAATAGG	TTAAGCGAGCCCTATACTTATG	60	180-188	Stolle et al., 2011
0867_70k14	FQ377846	B12	ATATTACATTCCTGGTGACCTC	CTACATTCTTTCTGTTCCCTGT	60	177-181	Stolle et al., 2011
0869_70d5	FQ377847	B08	ATCTGATATCTATGCGCTCTTT	AAGCAGATGGGTTAAGTGTAGT	60	163-175	Stolle et al., 2011
0885_52p13	FQ377850	B02	TTCATACTCTTTCACAGCCTCT	AATGACGAGATGAGACTGAAAT	60	160-164	Stolle et al., 2011
0887_52l8	FQ377851	B09	GGCGAGTGTAACGTTGTATTT	GATATTACGCTCTGGAACCAA	60	187-191	Stolle et al., 2011
0904_31d21	FQ377854	B14	TTAAACCGAGGAGAGAGATTAC	GAGAAGAGACGTTTGAGAGAAC	60	204-212	Stolle et al., 2011
0916_31f17	FQ377858	B06	CCCATCAAATTTAACTGTTCTT	GCGAGTCATTACTGTCTCTCTT	60	170	Stolle et al., 2011
0917_31j16	FQ377859	B16	GTGTGGAAGAGACGAGATAGAT	CTTCTTCGTCACGTTTACTCTC	60	188-204	Stolle et al., 2011
0919_66k13	FQ377861	B01	TAGACCGATTTGTTACTGATTG	CATGCTGTTATGGTATTTCTGA	60	164	Stolle et al., 2011
0930_40o1	FQ377864	B11	GCTGAAAGCTCGACTTCTAC	AAATTTCTCACTGCTAAGAGGA	60	157-177	Stolle et al., 2011
0939_33h17	FQ377865	B01	GAACACAGCGAGAAAGAGAG	CATTATCGTGTGAACTTGGAC	60	152-174	Stolle et al., 2011
0940_33f14	FQ377866	B02	AGTGGAAATCTCACACATGC	AGGAGTTTCGTCGTTTCTTT	60	175-177	Stolle et al., 2011
0942_23k3	FQ377867	B06	TCTCATTTCTCTCCTTCTTCC	ATACAAGAAACGAGCCAGATAC	60	223-225	Stolle et al., 2011

Marker	Accession number (GenBank)	LG *B.t.*	Forward primer sequence	Reverse primer sequence	*Ta*	Range	Source
0950_23a2	FQ377870	B18	CGTACTAAACGGTGTATCGTC	GTAATTGAGCTCTCCTGTGG	60	182	Stolle et al., 2011
0956_26c17	FQ377871	B02	TCTCTTGCTTCTCGTTCTACTT	GGCTCTTAAACCAGACAGTTT	60	173-175	Stolle et al., 2011
Apis_UN075	AADG05004561	B06	GTCGTCGCATGAAAGGCC	GCAACCTCGTGCCCAGAT	0	0	Solignac et al., 2007
B118		B03	CCTAAGTCGCTATATCTTCG	GAAACACGTATCTACATCTACAG	57	240	Estoup et al., (1993; 1995)
BL02		B01	GAACAGTGAGAGCGAGGAACAGAG	TTGCCACGTATATCCGAGCGAACC	52	163-171	Reber-Funk et al., (2006)
BL05		B07	CGTTCAACATTAGATGTAGAGTACC	CGGACACAAGTAATAAGATAGG	50	176-178	Reber-Funk et al., (2006)
BL13		B15	CGAATGTTGGGATTTTCGTG	GCGAGTACGTGTACGTGTTCTATG	53	205-217	Reber-Funk et al., (2006)
BL16		B13	CGTCCTCTCCAATGTGTGACTC	GGATCGGTTTAACAACGAAGTC	48	124-140	Reber-Funk et al., (2006)
Boro115	HQ682231	B06	AGGAACCGAGCGATAGAACCAC	GCTTTGCCTTTCCATCTTGCTG	47	175-183	Stolle et al., 2011
BT02		B11	AGGAACCGAGCGATAGAACCAC	GCTTTGCCTTTCCATCTTGCTG	53	175-183	Reber-Funk et al., (2006)
BT05		B02	TTTCCTATGCCGAACGTCACC	CCCAGATAAAAGACCGCCTCTAGTC	53	194-220	Reber-Funk et al., (2006)
BT08		B03	AGAACCTCCGTATCCCTTCG	AGCCTACCCAGTGCTGAAAC	52	208-230	Reber-Funk et al., (2006)
BT10		B08	TCTTGCTATCCACCACCCGC	GGACAGAAGCATAGACGCACCG	53	178-188	Reber-Funk et al., (2006)
BT11		B09	AAGAGAGAGACAGAGAGAGATAGGG	GCGTTTTGACGATTAGATTAGAGCC	52	153-177	Reber-Funk et al., (2006)
BT15		B15	ACTTAGCCAGCCATCGCTAC	CTCTCTCTTTCTCTCTCTTATACGC	53	182-214	Reber-Funk et al., (2006)
BT20		B10	TTCCACAGCGTTTTCTTAAGTC	ATGGACGGCGAGATCGTGAG	52	157-165	Reber-Funk et al., (2006)
BT23		B11	GCAACAGAAAATCGTCGGTAGTG	GCGGCAATAAAGCAATCGG	54	198-216	Reber-Funk et al., (2006)
BT24		B07	TCTTTCCGTTTTCCCCCTG	CACCCACTTACATACATACACGCTC	52	227-257	Reber-Funk et al., (2006)
BT30		B12	ATCGTATTATTGCCACCAACCG	CAGCAACAGTCACAACAAACGC	53	201-203	Reber-Funk et al., (2006)
BTERN01		B11	CGTGTTTAGGGTACTGGTGGTC	GGAGCAAGAGGGCTAGACAAAAG	49	120-122	Reber-Funk et al., (2006)
BTMS0071	FJ616203	B15	CGCGTAAATTATTCCCCTCCC	CCATCTCGCGCAGAATGTTT	57	237-239	Stolle et al., (2009)
BTMS0081	FJ616212	B06	ACGCGCGCCTTCTACTATC	AGGGACACGCGAACAGAC	60	321-327	Stolle et al., (2009)
BTMS0087	FJ616218	B10	CGCGACGTATAGACAGAGGA	AGCTGCCAGCGCTAAAGTAT	60	202-210	Stolle et al., (2009)
BTMS0093	FJ616224	B09	AGATTGCGATGGCTAAAGTCG	AAAGTCTCTACTGTCGCGCT	51	316-320	Stolle et al., (2009)
BTMS0099	FJ616230	B06	TGTCGGTGTTTCAACACTTTGT	AAAGAGGCGACTACGGTCAA	51	192-196	Stolle et al., (2009)
BTMS0102	FJ616233	B12	AATCGCAAGGGAAAACGTCC	TCTTCTCCGGTGTTTTCGGA	60	219-225	Stolle et al., (2009)
BTMS0103	FJ616234	B15	CAGGTGTTGCCGGCTAGATA	CTCAACGGATCTGGGACAGT	55	314-343	Stolle et al., (2009)
BTMS0124	FJ616254	B06	CGCCGTAATGTTAACTCC	ACTCAATCCAAACGCCACC	54	270	Stolle et al., (2009)

Marker	Accession number (GenBank)	LG *B.t.*	Forward primer sequence	Reverse primer sequence	*Ta*	Range	Source
BTMS0129	FJ616259	B10	CCTCGCGAATAGATAAAT	AGCTACCGTGCCTGTCC	55	154-160	Stolle et al., (2009)
BTMS0130	FJ616260	B09	AGACAAAGGGAGATGGTG	TTTCGTTCCTCGTGCTAC	52	302-306	Stolle et al., (2009)
BTMS0131	FJ616261	B10	TACAAACGATGCGTGAGG	AGTCAAGTAAGTCCTACCG	48	331-335	Stolle et al., (2009)
BTMS0147	FJ616276	B15	TTGAGAAAGTAGAAAAATGGA	TCTGTTTATCGATCCTCTTC	51	170-174	Stolle et al., (2009)
