# Supplementary material for: Quantitative Trait Loci for Light Sensitivity, Body Weight, Body Size, and Morphological Eye Parameters in the Bumblebee, Bombus terrestris
Source: PLoS One. 2015 Apr 30;10(4):e0125011. doi: 10.1371/journal.pone.0125011 (PMC4415782; doi:10.1371/journal.pone.0125011)
Supplement: S2 Table — The number of markers on each linkage group (n), the size of this linkage group (size LG), and the minimum (Min. d) and maximum (Max. d) distances between two markers on each linkage group. (PDF) [file pone.0125011.s003.pdf]

Quantitative trait loci for light sensitivity, body weight, body size, and morphological eye parameters in the bumblebee, *Bombus terrestris*

Kevin Maebe[1], Ivan Meeus[1], Jan De Riek[2], Guy Smagghe[1,*]

S2_Table: Distribution information of the 100 markers used for preliminary linkage mapping. The number of markers on each linkage group (n), the size of this linkage group (size LG), and the minimum (Min. d) and maximum (Max. d) distances between two markers on each linkage group.

	**size LG (cM)**	**n**	**Min. d (cM)**	**Max. d (cM)**
**LG01**	121.01	6	6.95	22.29
**LG02**	125.20	6	8.23	26.05
**LG03**	96.35	7	7.18	18.26
**LG04**	80.66	4	3.68	25.33
**LG05**	102.84	5	12.93	22.95
**LG06**	171.70	9	2.72	65.56
**LG07**	161.43	8	5.26	49.87
**LG08**	91.64	6	2.58	17.91
**LG09**	109.48	6	8.30	23.70
**LG10**	126.46	7	10.42	28.13
**LG11**	116.30	7	12.09	28.72
**LG12**	111.39	7	9.78	20.00
**LG13**	105.74	5	11.97	22.53
**LG14**	73.44	4	7.12	26.03
**LG15**	96.55	5	13.22	36.44
**LG16**	77.87	3	9.97	40.38
**LG17**	83.14	3	17.98	40.55
**LG18**	51.01	2	6.01	45.00
Mean	*105.68*	*5.56*	*8.69*	*31.09*
