# Supplementary material for: Quantitative Trait Loci for Light Sensitivity, Body Weight, Body Size, and Morphological Eye Parameters in the Bumblebee, Bombus terrestris
Source: PLoS One. 2015 Apr 30;10(4):e0125011. doi: 10.1371/journal.pone.0125011 (PMC4415782; doi:10.1371/journal.pone.0125011)
Supplement: S3 Table — (PDF) [file pone.0125011.s004.pdf]

Quantitative trait loci for light sensitivity, body weight, body size, and morphological eye parameters in the bumblebee, *Bombus terrestris*

Kevin Maebe[1], Ivan Meeus[1], Jan De Riek[2], Guy Smagghe[1,*]

S3_Table: Kolmogorov-Smirnov test of normality for each trait.

	Kolmogorov-Smirnov[a]		
**Trait**	**Statistic**	**df**	**Sig.**
RC	0.091	87	0.071
MT_L	0.088	87	0.095
MT_B	0.102	87	0.026
Ti_L	0.094	87	0.057
TI_B	0.053	87	0.200
Fe_L	0.119	87	0.004
Fe_B	0.300	87	0.000
Tr_L	0.074	87	0.200
Tr_B	0.120	87	0.004
Tarsus	0.092	87	0.066
Poot	0.104	87	0.021
E_L	0.124	87	0.002
E_B	0.186	87	0.000
Facet	0.135	87	0.000
Ocel	0.077	87	0.200
E_S	0.147	87	0.000
Omma	0.102	87	0.027
BLUE	0.271	87	0.000
UV	0.177	87	0.000
Weight	0.060	87	0.200
log_blue	0.174	87	0.000
log_uv	0.166	87	0.000
Pc1_E	0.134	87	0.001
Pc2_E	0.152	87	0.000
Pc3_E	0.066	87	0.200
Pc1_S	0.081	87	0.200
Pc2_S	0.131	87	0.001
Pc3_S	0.176	87	0.000
Pc4_S	0.066	87	0.200
Pc5_S	0.150	87	0.000
