# Supplementary material for: Quantitative Trait Loci for Light Sensitivity, Body Weight, Body Size, and Morphological Eye Parameters in the Bumblebee, Bombus terrestris
Source: PLoS One. 2015 Apr 30;10(4):e0125011. doi: 10.1371/journal.pone.0125011 (PMC4415782; doi:10.1371/journal.pone.0125011)
Supplement: S4 Table — The eigenvalues and eigenvectors of the PCA are given for the different body size traits and the eye parameters. (PDF) [file pone.0125011.s005.pdf]

Quantitative trait loci for light sensitivity, body weight, body size, and morphological eye parameters in the bumblebee, *Bombus terrestris*

Kevin Maebe[1], Ivan Meeus[1], Jan De Riek[2], Guy Smagghe[1,*]

S4_Table: PCA of the different body size traits and eye parameters. The eigenvalues and eigenvectors of the Principal Component Analysis of: (i) the different body size traits and (ii) the eye parameters.

(i) Principal Component Analysis (PCA): ***body size parameters***

Eigenvalues

PC	Eigenvalues	%Variation	Cum.%Variation
1	5,91	53,8	53,8
2	1,57	14,2	68,0
3	0,996	9,1	77,1
4	0,716	6,5	83,6
5	0,656	6,0	89,5

Eigenvectors

(Coefficients in the linear combinations of variables making up PC's)

Variable	PC1	PC2	PC3	PC4	PC5
Radial cell	-0,239	-0,179	-0,129	0,783	-0,395
Mt_L	-0,373	-0,085	-0,189	0,053	0,055
Mt_W	-0,362	-0,092	-0,173	-0,205	0,126
Ti_L	-0,395	-0,044	-0,153	-0,043	0,070
Ti_W	-0,346	-0,101	-0,179	-0,313	-0,081
Fe_L	-0,356	-0,049	-0,311	-0,113	-0,055
Fe_W	-0,210	-0,130	0,575	-0,218	-0,681
Tr_L	-0,225	-0,319	0,461	-0,162	0,285
Tr_W	-0,250	-0,104	0,430	0,391	0,516
Tarsus	-0,239	0,635	0,135	0,045	0,005
Leg	-0,237	0,636	0,136	0,029	-0,015

(ii) Principal Component Analysis (PCA): ***eye parameters***

Eigenvalues

PC	Eigenvalues	%Variation	Cum.%Variation
1	4,45	74,1	74,1
2	0,654	10,9	85,0
3	0,488	8,1	93,2

Eigenvectors

(Coefficients in the linear combinations of variables making up PC's)

Variable	PC1	PC2	PC3
E_L	-0,458	-0,013	0,118
E_W	-0,456	-0,021	0,113
Facet	-0,325	0,829	-0,319
MOc	-0,388	-0,235	0,521
E_S	-0,453	0,030	0,134
Om	-0,347	-0,506	-0,763
