# Supplementary material for: Quantitative Trait Loci for Light Sensitivity, Body Weight, Body Size, and Morphological Eye Parameters in the Bumblebee, Bombus terrestris
Source: PLoS One. 2015 Apr 30;10(4):e0125011. doi: 10.1371/journal.pone.0125011 (PMC4415782; doi:10.1371/journal.pone.0125011)
Supplement: S5 Table — List of the place, accession number, name and annotation information of all genes, at suggestive QTL qBLU3 on LG 3, which can all be linked with the critical light sensitivity of bumblebee drones in blue light. (PDF) [file pone.0125011.s006.pdf]

Quantitative trait loci for light sensitivity, body weight, body size, and morphological eye parameters in the bumblebee, *Bombus terrestris*

Kevin Maebe[1], Ivan Meeus[1], Jan De Riek[2], Guy Smagghe[1,*]

S5_Table: List of candidate genes for critical light sensitivity of bumblebee drones in blue light. List of the place, accession number, name and annotation information of all genes, at suggestive QTL qBLU3 on LG 3, which can all be linked with the critical light sensitivity of bumblebee drones in blue light.

start	stop	Accession	Locus	Description
1379268	1440568	XM_003394234.1	LOC100651751	sex-regulated protein janus-A-like (LOC100651751), mRNA
1380872	1425353	XM_003394227.1	LOC100650954	phosrestin-1-like (LOC100650954), mRNA
1444315	1449818	XM_003394350.1	LOC100646286	hypothetical protein LOC100646286 (LOC100646286), mRNA
1450487	1452349	XM_003394349.1	LOC100646173	hypothetical protein LOC100646173 (LOC100646173), mRNA
1500499	1526455	XM_003394228.1	LOC100651071	hypothetical protein LOC100651071 (LOC100651071), mRNA
1563777	1575057	XM_003394224.1	LOC100650594	probable ATP-dependent RNA helicase CG8611-like (LOC100650594), mRNA
1574645	1588141	XM_003394225.1	LOC100650718	hypothetical protein LOC100650718 (LOC100650718), mRNA
1592463	1595546	XM_003394223.1	LOC100650476	integrator complex subunit 10-like (LOC100650476), mRNA
1594866	1601254	XM_003394221.1	LOC100650237	protein transport protein Sec31A-like (LOC100650237), mRNA
1601991	1605404	XM_003394220.1	LOC100650112	polypeptide N-acetylgalactosaminyltransferase 3-like (LOC100650112), mRNA
1612411	1629559	XM_003394348.1	LOC100646056	hypothetical protein LOC100646056 (LOC100646056), mRNA
1740947	1796898	XM_003394219.1	LOC100649991	transcription factor hamlet-like (LOC100649991), mRNA
1965450	1986889	XM_003394347.1	LOC100645939	SPRY domain-containing SOCS box protein 1-like (LOC100645939), mRNA
1994868	1996819	XM_003394346.1	LOC100645823	hypothetical protein LOC100645823 (LOC100645823), mRNA
2007601	2024300	XM_003394218.1	LOC100649874	cyclin-dependent kinase 5 activator 1-like (LOC100649874), mRNA
2028512	2029978	XM_003394217.1	LOC100649763	protein transport protein SFT2-like (LOC100649763), mRNA
2029943	2031725	XR_131866.1	LOC100649651	hypothetical LOC100649651 (LOC100649651), miscRNA
2033121	2041004	XM_003394216.1	LOC100649528	hypothetical protein LOC100649528 (LOC100649528), mRNA
2048620	2057193	XM_003394215.1	LOC100649406	serine/threonine-protein phosphatase 4 regulatory subunit 4-like (LOC100649406), mRNA
2058202	2237814	XM_003394213.1	LOC100649165	hypothetical protein LOC100649165 (LOC100649165), mRNA
2154619	2155506	XM_003394214.1	LOC100649286	hypothetical protein LOC100649286 (LOC100649286), mRNA
2239904	2242915	XM_003394344.1	LOC100645588	hypothetical protein LOC100645588 (LOC100645588), mRNA

start	stop	Accession	Locus	Description
2242543	2245756	XM_003394212.1	LOC100649049	kelch domain-containing protein 10-like (LOC100649049), mRNA
2250046	2266693	XR_131865.1	LOC100648815	hypothetical LOC100648815 (LOC100648815), miscRNA
2265285	2270449	XM_003394211.1	LOC100648931	hypothetical protein LOC100648931 (LOC100648931), mRNA
2270494	2271893	XM_003394210.1	LOC100648703	DNA repair protein RAD51 homolog 4-like (LOC100648703), mRNA
2275283	2295240	XM_003394209.1	LOC100648591	hypothetical protein LOC100648591 (LOC100648591), mRNA
2297014	2299967	XM_003394208.1	LOC100648391	cytochrome P450 6k1-like (LOC100648391), mRNA
2299671	2305966	XM_003394207.1	LOC100648275	DNA topoisomerase 2-binding protein 1-like (LOC100648275), mRNA
2306495	2489235	XM_003394206.1	LOC100648160	protein-tyrosine sulfotransferase-like (LOC100648160), mRNA
2490288	2494623	XM_003394205.1	LOC100648049	protein MTO1 homolog, mitochondrial-like (LOC100648049), mRNA
2499310	2650532	XM_003394204.1	LOC100647927	probable G-protein coupled receptor Mth-like 1-like (LOC100647927), mRNA
2668080	2681265	XM_003394203.1	LOC100647804	hypothetical protein LOC100647804 (LOC100647804), mRNA
2720952	2732569	XM_003394202.1	LOC100647689	GTP-binding protein Rhes-like (LOC100647689), mRNA
2733853	2735909	XM_003394201.1	LOC100647569	hypothetical protein LOC100647569 (LOC100647569), mRNA
2811892	2813218	XM_003394343.1	LOC100645483	somatostatin receptor type 4-like (LOC100645483), mRNA
2871628	2940085	XM_003394199.1	LOC100647330	guanylate cyclase 32E-like (LOC100647330), mRNA
2939790	2940990	XM_003394200.1	LOC100647452	NADH dehydrogenase [ubiquinone] 1 alpha subcomplex subunit 6-like (LOC100647452), mRNA
2941318	2944004	XM_003394198.1	LOC100647213	tRNA-splicing ligase RtcB homolog (LOC100647213), mRNA
2944108	3059184	XR_131874.1	LOC100645366	hypothetical LOC100645366 (LOC100645366), miscRNA
3062781	3065425	XM_003394195.1	LOC100646894	pescadillo homolog (LOC100646894), mRNA
3065480	3070181	XM_003394194.1	LOC100646771	protein pelota-like (LOC100646771), mRNA
3070000	3073295	XM_003394196.1	LOC100647012	probable serine hydrolase-like, transcript variant 1 (LOC100647012), mRNA
3070000	3074784	XM_003394197.1	LOC100647012	probable serine hydrolase-like, transcript variant 2 (LOC100647012), mRNA
3075349	3076615	XM_003394193.1	LOC100646650	hypothetical protein LOC100646650 (LOC100646650), mRNA
3076597	3081931	XM_003394192.1	LOC100646529	UPF0636 protein C4orf41 homolog (LOC100646529), mRNA
3082052	3084507	XM_003394191.1	LOC100646403	probable G-protein coupled receptor AH9.1-like (LOC100646403), mRNA
3086097	3087571	XM_003394342.1	LOC100645238	lymphokine-activated killer T-cell-originated protein kinase-like (LOC100645238), mRNA
3087641	3094415	XM_003394190.1	LOC100646285	syntaxin-1A homolog (LOC100646285), mRNA
3088939	3095239	XM_003394189.1	LOC100646172	condensin-2 complex subunit D3-like (LOC100646172), mRNA
3097469	3108358	XM_003394188.1	LOC100646055	ankyrin repeat domain-containing protein 29-like (LOC100646055), mRNA
3108776	3118908	XM_003394187.1	LOC100645904	acyl carrier protein, mitochondrial-like (LOC100645904), mRNA
3109468	3117237	XM_003394185.1	LOC100645705	TBC1 domain family member 9-like, transcript variant 1 (LOC100645705), mRNA
3109535	3116937	XM_003394186.1	LOC100645705	TBC1 domain family member 9-like, transcript variant 2 (LOC100645705), mRNA
3119005	3122275	XM_003394182.1	LOC100645365	mps one binder kinase activator-like 4-like (LOC100645365), mRNA
3121758	3138923	XM_003394183.1	LOC100645482	alba-like protein C9orf23 homolog (LOC100645482), mRNA

start	stop	Accession	Locus	Description
3127235	3130577	XM_003394180.1	LOC100644997	aromatic-L-amino-acid decarboxylase-like (LOC100644997), mRNA
3133043	3137030	XM_003394181.1	LOC100645237	histidine decarboxylase-like (LOC100645237), mRNA
3137421	3138388	XM_003394184.1	LOC100645587	peptidyl-prolyl cis-trans isomerase H-like (LOC100645587), mRNA
3139162	3142194	XR_131864.1	LOC100645116	zinc finger protein 595-like (LOC100645116), miscRNA
3142893	3146252	XM_003394179.1	LOC100644877	cGMP-dependent protein kinase, isozyme 1-like (LOC100644877), mRNA
3146544	3170527	XR_131863.1	LOC100644751	ATP-binding cassette sub-family C member 9-like (LOC100644751), miscRNA
3178322	3510902	XM_003394178.1	LOC100644512	RNA binding protein fox-1 homolog 2-like (LOC100644512), mRNA
3178340	3363539	XM_003394177.1	LOC100644512	RNA binding protein fox-1 homolog 2-like (LOC100644512), mRNA
